# Structural basis for extracellular *cis* and *trans* RPTPσ signal competition in synaptogenesis

**DOI:** 10.1038/ncomms6209

**Published:** 2014-11-11

**Authors:** Charlotte H. Coles, Nikolaos Mitakidis, Peng Zhang, Jonathan Elegheert, Weixian Lu, Andrew W. Stoker, Terunaga Nakagawa, Ann Marie Craig, E. Yvonne Jones, A. Radu Aricescu

**Affiliations:** 1 Division of Structural Biology, Wellcome Trust Centre for Human Genetics, University of Oxford, Roosevelt Drive, Oxford OX3 7BN, UK; 2 Brain Research Centre and Department of Psychiatry, University of British Columbia, Vancouver, British Columbia, Canada V6T 2B5; 3 Cancer Section, Institute of Child Health, University College London, 30 Guilford Street, London WC1N 1EH, UK; 4 Department of Molecular Physiology and Biophysics, Vanderbilt University, School of Medicine, 702 Light Hall (0615), Nashville, Tennessee 37232-0615, USA

## Abstract

Receptor protein tyrosine phosphatase sigma (RPTPσ) regulates neuronal extension and acts as a presynaptic nexus for multiple protein and proteoglycan interactions during synaptogenesis. Unknown mechanisms govern the shift in RPTPσ function, from outgrowth promotion to synaptic organization. Here, we report crystallographic, electron microscopic and small-angle X-ray scattering analyses, which reveal sufficient inter-domain flexibility in the RPTPσ extracellular region for interaction with both *cis* (same cell) and *trans* (opposite cell) ligands. Crystal structures of RPTPσ bound to its postsynaptic ligand TrkC detail an interaction surface partially overlapping the glycosaminoglycan-binding site. Accordingly, heparan sulphate and heparin oligomers compete with TrkC for RPTPσ binding *in vitro* and disrupt TrkC-dependent synaptic differentiation in neuronal co-culture assays. We propose that transient RPTPσ ectodomain emergence from the presynaptic proteoglycan layer allows capture by TrkC to form a trans-synaptic complex, the consequent reduction in RPTPσ flexibility potentiating interactions with additional ligands to orchestrate excitatory synapse formation.

## Abstract (web summary)

Receptor protein tyrosine phosphatase sigma (RPTPσ) promotes both neurite outgrowth and synaptic organization. Here, Coles *et al.* present the structural basis for this switch in function, whereby TrkC on the postsynaptic membrane and heparan sulphate proteoglycans compete for the same binding surface on RPTPσ.

## Graphical abstract

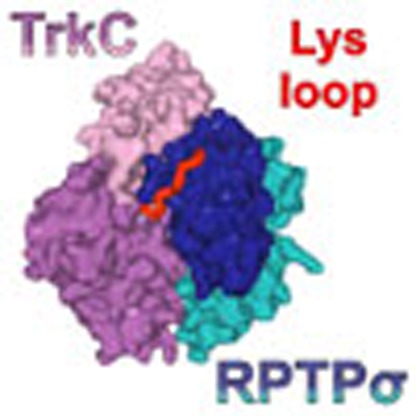

Neuronal synaptogenesis is orchestrated by cell surface receptors, which define the formation and functionality of distinct synapse classes^1^,^2^. These ‘organizer’ molecules can act as scaffolds or hubs, to integrate multiple inputs into a unified cellular response. This concept is well established for intracellular systems, but only now emerging for cell surface molecules such as neurexin-neuroligin and repulsive guidance molecule (RGM)-neogenin protein complexes^3^,^4^,^5^. At the axonal surface, type IIa receptor protein tyrosine phosphatases (RPTPs) (type IIa RPTPs, for example, RPTPσ, RPTPδ and leukocyte common antigen-related (LAR) in vertebrates, dLAR in *Drosophila*), are a recently identified nexus for extracellular interactions, receiving signals and transmitting them intracellularly to the cytoskeleton to regulate neuronal extension and guidance, as well as synaptic organization^6^,^7^,^8^,^9^.

Heparan sulphate proteoglycans (HSPGs) and chondroitin sulphate proteoglycans (CSPGs) play important roles in the modulation of RPTP signalling in the nervous system^10^,^11^,^12^,^13^,^14^,^15^. At the *Drosophila* neuromuscular junction, the HSPGs dSyndecan and dDallylike opposingly regulate dLAR-mediated synaptic morphogenesis and active zone function^12^. While the interaction of dLAR with presynaptic dSyndecan promotes bouton growth, postsynaptic dDallylike competes with dSyndecan for dLAR binding, leading to an inhibition of growth and active zone stabilization^12^. Interactions of the RPTPσ ectodomain with HSPGs and CSPGs modulate axonal growth both during development and post injury^10^,^13^,^14^,^16^. HSPGs cluster RPTPσ, a characteristic proposed to drive a localized imbalance of protein tyrosine phosphorylation and hence promote growth^14^. Once the axon has reached its final target, RPTPσ can establish direct interactions with multiple postsynaptic proteins. In vertebrates, these include the TrkC receptor protein tyrosine kinase, Netrin-G ligand-3 (NGL-3), interleukin-1 receptor accessory protein and Slit- and Trk-like receptors 1 and 2 (Slitrk1 and Slitrk2)^17^,^18^,^19^,^20^. These trans-synaptic complexes mediate bi-directional excitatory synapse formation, simultaneously triggering presynaptic differentiation and an accumulation of synaptic vesicles, and clustering of the postsynaptic density^7^,^9^.

There are two major neuronal RPTPσ isoforms, sharing a common intracellular catalytic region and an extracellular region predicted to contain three immunoglobulin (Ig)-like domains followed by either five or nine fibronectin (FN) type III domains, in the central and peripheral nervous systems, respectively^21^. Further isoforms include a combination of four mini-exons (meA-meD) that may modulate interactions with protein partners (Fig. 1a)^8^,^21^. Previous mutagenesis and structural studies have demonstrated that the proteoglycan-binding site lies on Ig1 of RPTPσ, and comprises an extended positively charged surface of basic residues^10^,^13^,^14^. Binding of postsynaptic TrkC, is reported to require the N-terminal three Ig domains of RPTPσ^17^; the NGL-3-binding site has been mapped to the FN1-2 domains^18^.

The properties that fit the RPTPσ ectodomain for its function as an integrative hub for signalling in synaptogenesis are unknown. Here we report a molecular level analysis of the RPTPσ ectodomain and of its direct interactions with the postsynaptic binding partner TrkC. We reveal that the multi-domain extracellular region of RPTPσ is unexpectedly flexible. This characteristic confers sufficient conformational freedom to allow its binding to both pre- or postsynaptic ligands. RPTPσ:TrkC crystal structures provide an explanation for the specificity of this interaction and also highlight an overlap of TrkC and proteoglycan-binding sites on RPTPσ. This observation suggests that there is competition between TrkC and heparan sulphate for RPTPσ binding, and we provide support for this notion in biophysical and cellular assays. Overall, our study provides novel insights into the mechanisms determining the hierarchy and functional consequences of RPTPσ–ligand interactions.

## Results

### The RPTPσ ectodomain exhibits extensive flexibility

**Figure 1:**
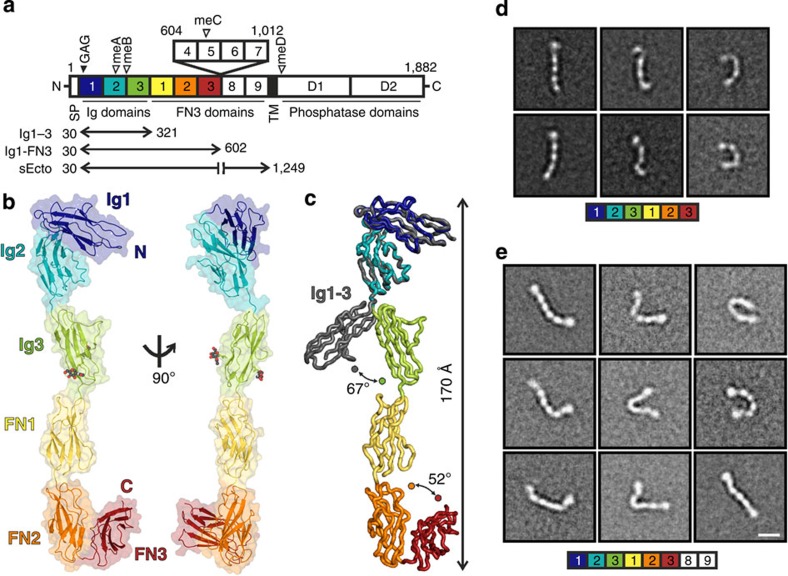
RPTPσ ectodomain flexibility. (**a**) RPTPσ domain organization. N, amino-terminus (extracellular); SP, secretion signal peptide; TM, transmembrane; C, C terminus (intracellular); Ig, immunoglobulin-like; FN, fibronectin type-III; GAG, glycosaminoglycan-binding site (filled arrowhead). Alternative splicing inserts: FN domains 4–7 and mini-exons A–D (open arrowhead). (**b**) Ribbon and surface representations of the human RPTPσ Ig1-FN3 crystal structure. *N*-linked glycans in atom representation. (**c**) Ig3 movement in Ig1-FN3 relative to Ig1–3 (grey, PDB ID: 2YD9) structure. Representative RPTPσ Ig1-FN3 (**d**) and RPTPσ sEcto (**e**) negative-stain electron microscopy class averages. Scale bar, 10 nm. Full sets of RPTPσ Ig1-FN3 and sEcto class averages are provided in Supplementary Figs 2 and 3.

We first investigated the molecular characteristics of RPTPσ that allow presentation of the N-terminal Ig domains for both pre- and postsynaptic ligand binding. We determined the 3.15 Å crystal structure of a six N-terminal domain human RPTPσ construct (termed Ig1-FN3, Fig. 1a), which contains all the binding sites for synaptic ligands identified to date. This construct maintains the V-shaped Ig1–2 arrangement previously reported^14^,^22^, followed by an extended conformation of the sequential domains Ig3, FN1 and FN2 (Fig. 1b; Table 1). Superposition of Ig1-FN3 with the crystal structure of Ig1–3 (ref. 14) revealed a hinge point between domains Ig2 and Ig3 (Fig. 1c). The four amino-acid meB exon would extend this apparently flexible linker further, by four residues (Supplementary Fig. 1). While FN1 and FN2 domains align approximately with the long axis of the molecule, the C-terminal FN3 domain folds back, suggesting the FN2-3 linker may also be a flexion point. Since no substantial Ig3-FN1, FN1–FN2 or FN2–FN3 inter-domain interfaces are apparent, we hypothesized that when released from crystal packing constraints each of the Ig2-FN3 region linkers may provide substantial flexibility. To test this, single-particle negative-stain electron microscopy (EM) class averages of human RPTPσ Ig1-FN3 were calculated. These showed a broad range of conformations (Fig. 1d; Supplementary Fig. 2). This flexibility, reminiscent of hinge points in α-neurexin^23^, is in marked contrast to the rigidity of homophilic cell adhesion molecules of similar size and domain organization, such as cadherins, RPTPμ and SYG-1/SYG-2 (refs 24, 25, 26).

We extended our EM analysis to the full ectodomain of the eight-domain isoform of human RPTPσ (sEcto; Fig. 1a,e; Supplementary Fig. 3). The 150 class averages generated reinforce our conclusions from the six-domain structural analyses. The RPTPσ ectodomain exhibits a surprisingly large flexibility, with observed conformations ranging from almost fully extended, to essentially bent double (Fig. 1e; Supplementary Fig. 3). To control for the potential risk of artefacts associated with negative staining, we also performed small-angle X-ray scattering (SAXS) measurements, at a physiological pH (7.4), for both human RPTPσ Ig1-FN3 and sEcto. This analysis further supports the observation that both proteins are flexible and are likely to adopt multiple conformations in solution (Supplementary Fig. 4). Taken together, crystallographic, EM and SAXS analyses demonstrate that the ectodomain of RPTPσ is able to explore a large conformational space.

### Structural analysis of the RPTPσ:TrkC trans-synaptic complex

**Figure 2:**
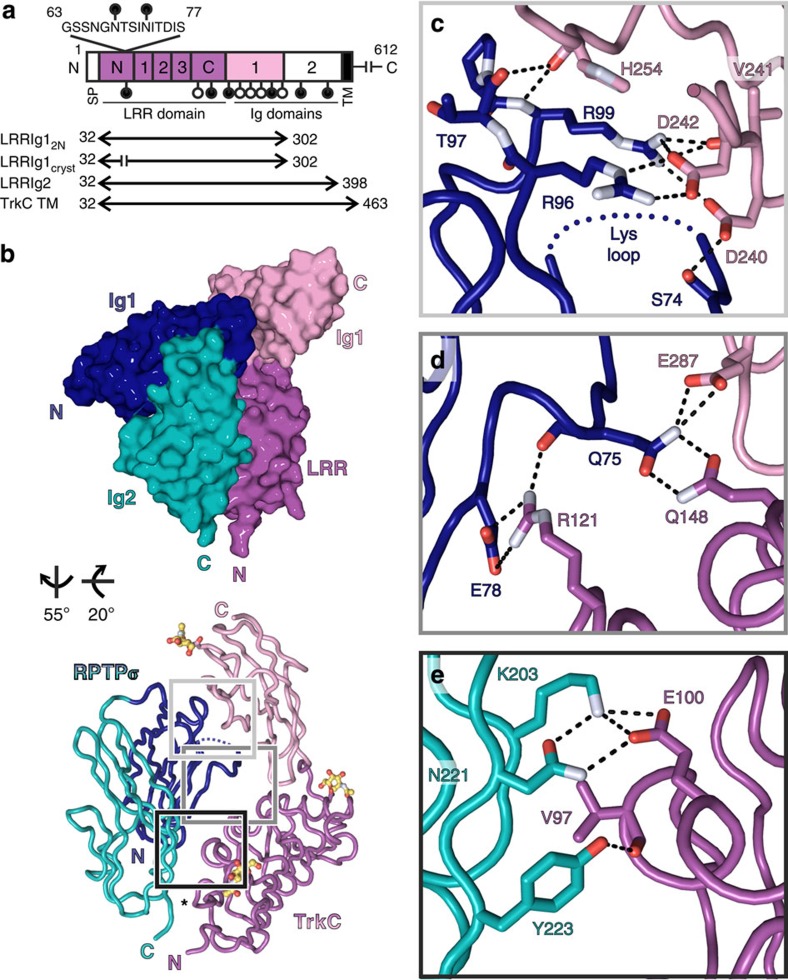
Trans-synaptic RPTPσ:TrkC complex crystal structure. (**a**) TrkCTK- (non-catalytic isoform) domain organization. LRR, leucine-rich repeat region (N, N-terminal cysteine-rich region; 1–3, leucine-rich repeats; C, C-terminal cysteine-rich region). Putative *N*-linked glycosylation sites, lollipops; filled lollipops remain in LRRIg1_cryst_ construct. (**b**) Space-filled and tube representations of chicken RPTPσ Ig1–2:TrkC LRRIg1_cryst_ crystal structure. *N*-linked glycans in atom representation. Disordered RPTPσ Lys-loop, blue dotted line; TrkC LRRIg1_cryst_ amino-acid residue 62–78 junction, asterisk. (**c**–**e**) Detailed view of bonding interactions at RPTPσ:TrkC interface for binding sites 1–3. Corresponding electron density is illustrated in Supplementary Fig. 7. Potential electrostatic and hydrogen bonds, black dashed lines; oxygen atoms, red; nitrogen atoms, bluewhite.

How do these conformational properties contribute to the interaction of RPTPσ with ligands? We sought to compare and contrast complex formation between the RPTPσ N-terminal region and axonal HSPGs^14^ with the trans-synaptic interaction between RPTPσ and TrkC. The TrkC ectodomain comprises an N-terminal leucine-rich repeat (LRR) domain, followed by two Ig domains (Fig. 2a). Equilibrium surface plasmon resonance (SPR) assays confirmed previously reported data^17^ that the minimal units required for full affinity binding are RPTPσ Ig1–3 and TrkC LRRIg1 (Supplementary Fig. 5a,b; Supplementary Table 1). RPTPσ Ig1–2 does retain TrkC binding, although with an approximately fivefold-reduced affinity compared with RPTPσ Ig1–3 (*K*_d_=2.4 μM versus 551 nM, Supplementary Fig. 5a). TrkC LRRIg1 had also previously been shown to be the minimal unit required for the synaptogenic activity of TrkC^17^. To facilitate crystallization, a chicken TrkC construct (TrkC LRRIg1_cryst_) was generated, which removed putative sites of *N*-linked glycosylation and residues 63–77, a predicted disordered loop (Supplementary Fig. 6). A 2.5Å crystal structure of a chicken RPTPσ Ig1–2:TrkC LRRIg1_cryst_ complex was determined, revealing a 1:1 stoichiometry (Fig. 2b; Table 1; Supplementary Fig. 5c), in agreement with results from multi-angle light scattering (MALS) analysis (Supplementary Fig. 5d). The structure is consistent with a *trans* RPTPσ:TrkC complex spanning the synaptic cleft (Fig. 2b). The V-shaped RPTPσ Ig1–2 module contacts an extended TrkC surface consisting of the LRR convex face and Ig1 domain, with a buried surface area of 1,093 Å^2^ per molecule (Fig. 2b).

Three major contact sites constitute the protein–protein interface in the complex crystal structure: site 1, RPTPσ Ig1:TrkC Ig1 (Fig. 2c; Supplementary Fig. 7); site 2, RPTPσ Ig1:TrkC LRRIg1 inter-domain region (Fig. 2d; Supplementary Fig. 7) and site 3, RPTPσ Ig2:TrkC LRR (Fig. 2e; Supplementary Fig. 7). Electrostatic interactions involving RPTPσ residues R96 and R99 and TrkC residues D240 and D242 dominate interactions at site 1 (Fig. 2c). Intriguingly, R96 and R99 form part of the extended positively charged surface on RPTPσ Ig1 (ref. 14) and are absolutely required for RPTPσ interactions with HSPGs^10^,^14^, suggesting that TrkC and proteoglycans may compete for binding to RPTPσ. At site 2, Q75 in RPTPσ, interacts with TrkC residues E287 and Q148, while the side chains of RPTPσ E78 and TrkC R121 form a salt bridge (Fig. 2d). Site 3 centres on a K203–E100 salt bridge between RPTPσ and TrkC residues, respectively (Fig. 2e).

**Figure 3:**
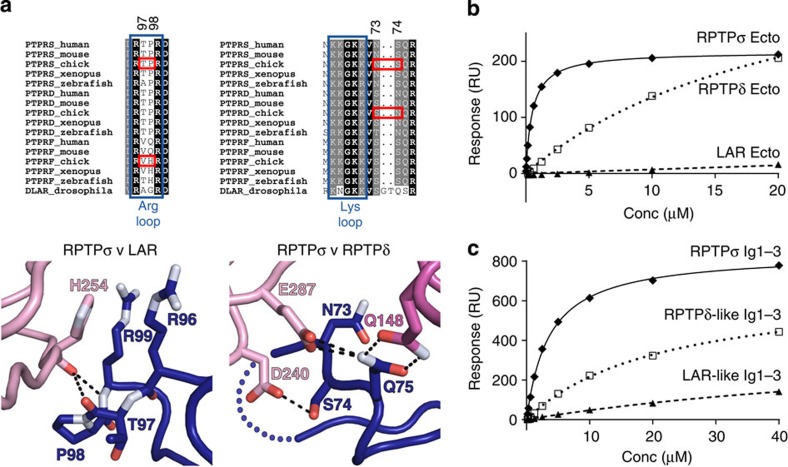
TrkC binding preferences for type IIa RPTP family members. (**a**) Type IIa RPTP sequence alignments and detailed views of the RPTPσ:TrkC crystal structure at binding site 1 (left) and binding site 2 (right). Blue boxes indicate RPTPσ residues required for proteoglycan binding, and red boxes highlight key sequence differences conferring TrkC-binding specificity. Colour scheme as in Fig. 2: dark blue, RPTPσ Ig1; pink, TrkC LRR; magenta, TrkC Ig1. (**b**) SPR analysis of human type IIa RPTP ectodomains binding to immobilized mouse TrkC LRRIg1. Measured binding values: RPTPσ Ecto, *K*_d_=516 nM and *B*_max_=217 RU; RPTPδ Ecto, *K*_d_>22 μM and *B*_max_>433 RU; RPTP LAR, *K*_d_ and *B*_max_ not determined. (**c**) SPR analysis of chicken TrkC LRRIg1 binding to immobilized chicken RPTPσ Ig1–3, RPTPσ N73S+S74N (RPTPδ-like) Ig1–3 and RPTPσ P97V+T98H (LAR-like) Ig1–3. Measured binding values: RPTPσ Ig1–3, *K*_d_=3.5 μM and *B*_max_=837 RU; RPTPδ-like Ig1–3, *K*_d_>21 μM and *B*_max_>674 RU; RPTP LAR-like Ig1–3, *K*_d_ and *B*_max_ not determined. For sensograms see Supplementary Fig. 8a,b.

These three interaction sites are consistent with RPTPσ binding TrkC, but not TrkA or TrkB^17^; of the six predominantly charged (D240, D242, E287, Q148, R121 and E100) TrkC residues observed to have side-chain-mediated RPTPσ interactions, only E287 is conserved across the other Trk family members (Supplementary Fig. 6). Although the key RPTPσ residues discussed above are conserved in human RPTPδ and LAR (Supplementary Fig. 1), the specificity of TrkC binding for RPTPσ can be rationalized through closer inspection of type IIa RPTP sequence alignments and the chicken RPTPσ Ig1–2:TrkC LRRIg1_cryst_ crystal structure (Fig. 3a). At site 1, we hypothesized that substitution of P98 (in RPTPσ) to H98 in RPTP LAR, would result in a movement of the carbonyl group of T97 to ease the *cis*-conformation of residue 98 to *trans* (see PDB ID: 2YD5), disrupting interaction with the TrkC H254 carbonyl group. At site 2, we anticipated that substitution of S74 (in RPTPσ) to N74 in RPTPδ would result in the loss of this residue’s interaction with TrkC D240. To validate these predictions, RPTPσ Ig1–3 P97V+T98H (LAR-like Ig1–3) and N73S+S74N (RPTPδ-like Ig1–3) proteins were generated, which indeed displayed LAR- and RPTPδ-like binding to TrkC in SPR analyses (Fig. 3b,c; Supplementary Fig. 8a,b; Supplementary Table 1).

### Validation of the RPTPσ:TrkC binding mode

**Figure 4:**
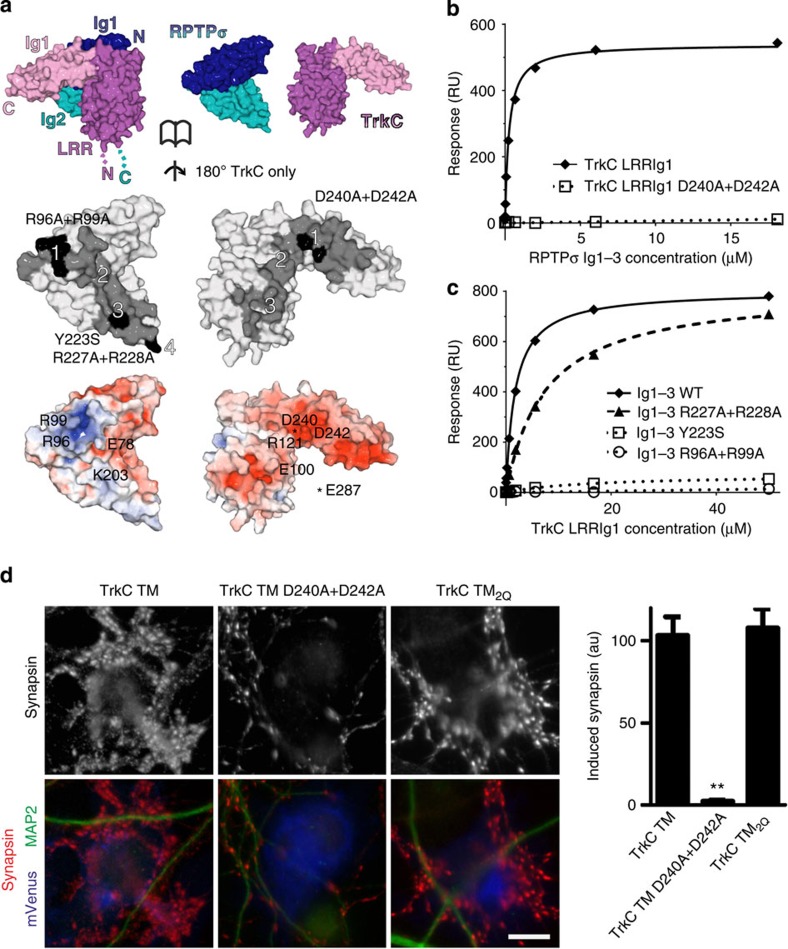
Validation of RPTPσ:TrkC binding interfaces. (**a**) Opened-view surface representation of the chicken RPTPσ Ig1–2:TrkC LRRIg1_cryst_ crystal structure. RPTPσ and TrkC interface residues are coloured grey and interface mutants used in biophysical and cellular assays are highlighted in black (middle panel). Binding sites 1–4 are labelled. RPTPσ and TrkC are coloured by electrostatic potential (bottom panel) from red (−8 kT/e) to blue (+8 kT/e), illustrating complementary charged patches at binding sites 1–3 (note that the basic RPTPσ ‘Lys-loop’ residues 68–71 are absent in the RPTPσ:TrkC complex crystallographic model). (**b**) SPR analysis of human RPTPσ Ig1–3 binding to immobilized mouse TrkC LRRIg1 and TrkC LRRIg1 D240A+D242A. Measured binding values: TrkC LRRIg1, *K*_d_=258 nM and *B*_max_=540 RU; TrkC LRRIg1 D240A+D242A, *K*_d_ and *B*_max_ not determined. (**c**) Mouse TrkC LRRIg1 binding to immobilized human RPTPσ Ig1–3 WT, R96A+R99A, Y223S and R227A+R228A. Measured binding values: RPTPσ Ig1–3 WT, *K*_d_=1.8 μM and *B*_max_=802 RU; Ig1–3 R227A+R228A, *K*_d_=7 μM and *B*_max_=806 RU; Ig1–3 R96A+R99A and Y223S, *K*_d_ and *B*_max_ not determined. For sensograms see Supplementary Fig. 8c–f. (**d**) Induced synapsin clustering in rat hippocampal neurons by TrkC TM (WT), TrkC TM_D240A+D242A and TrkC TM_2Q_ expressing COS-7 cells. Analysis of variance, *P*<0.0001; **P<0.001 compared with TrkC TM by *post hoc* Bonferroni’s multiple comparison test, *n*=26 cells from two experiments. Scale bar, 10 μM. Relative cell surface expression levels are shown in Supplementary Fig. 9.

To confirm the contribution of the RPTPσ:TrkC interaction sites, we introduced point mutations into the interfaces on either protein (Fig. 4a) and measured the resulting dissociation constants (*K*_d_) using SPR. As predicted, mutations in either TrkC (D240A+D242A) or RPTPσ (R96A+R99A, affecting the glycosaminoglycan (GAG)-binding arginine residues), completely abolished both human and chicken RPTPσ binding to mouse and chicken TrkC, respectively (Fig. 4b,c; Supplementary Fig. 8c–f; Supplementary Table 1). The RPTPσ Y223S mutation was designed to disrupt binding at site 3 by introducing an *N*-linked glycosylation site at N221, and RPTPσ:TrkC binding was indeed ablated (Fig. 4c; Supplementary Fig. 8e,f; Supplementary Table 1). In agreement with the structural and biophysical data, TrkC transmembrane (TrkC TM) D240A+D242A expressing COS-7 cells, unlike wild-type TrkC TM expressing positive controls, were unable to induce presynaptic differentiation in co-cultured rat hippocampal neurons despite comparable levels of cell surface expression (Fig. 4d; Supplementary Fig. 9).

**Figure 5:**
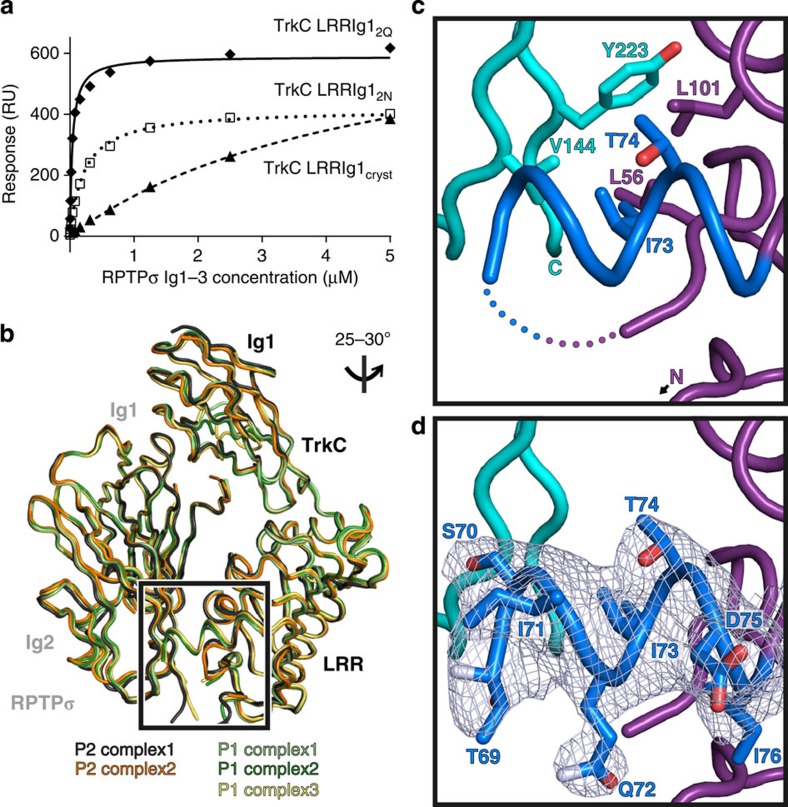
Characterization of the potential accessory RPTPσ:TrkC-binding site 4. (**a**) SPR analysis of chicken RPTPσ Ig1–3 binding to immobilized chicken TrkC LRRIg1_cryst_, LRRIg1_2N_ and LRRIg1_2Q_. Measured binding values: TrkC LRRIg1_cryst_, *K*_d_=4.8 μM and *B*_max_=761 RU; LRRIg1_2N_, *K*_d_=216 nM and *B*_max_=416 RU; LRRIg1_2Q_, *K*_d_=38 nM and *B*_max_=590 RU. For sensograms see Supplementary Fig. 8g–h. (**b**) Alignment of the two RPTPσ:TrkC complexes observed in the chicken RPTPσ Ig1–2:TrkC LRRIg1_cryst_ (P2 space group) and three in the chicken RPTPσ Ig1–3:TrkC LRRIg1_2Q_ (P1 space group) crystal structures. The orientation of the structures is identical to Fig. 2b (lower panel). (**c**) Additional features observed in complex 1 from the RPTPσ Ig1–3:TrkC LRRIg1_2Q_ crystal structure. Residues within the 63–77 loop that were not present in the P2 crystal structure are coloured blue and the remaining missing residues are indicated by dotted lines. View rotated relative to **b** as indicated. TrkC LRR, magenta; RPTPσ, cyan. (**d**) SigmaA weighted 2F_o_−F_c_ electron density map (grey) contoured at 1σ and carved at 2.2 Å around loop residues 69–76.

We had noted that the interaction affinity between engineered proteins used for RPTPσ:TrkC complex crystallization (chicken RPTPσ Ig1–3 and TrkC LRRIg1_cryst_) was some 20-fold lower following the TrkC 63–77 loop deletion (*K*_d_ 4.8 μM versus 216 nM; Fig. 5a; Supplementary Fig. 8g; Supplementary Table 1). We therefore explored the possible contribution of the 63–77 loop to the RPTPσ:TrkC interaction by engineering a construct termed TrkC LRRIg1_2Q_, comprising the full sequence but still removing two predicted *N*-linked glycosylation sites by introducing N68Q and N72Q mutations (Supplementary Fig. 6). This construct did indeed bind RPTPσ with enhanced affinity (Fig. 5a; Supplementary Fig. 8g,h; Supplementary Table 1), while TrkC TM_2Q_ induced comparable levels of presynaptic differentiation in co-cultured rat hippocampal neurons to wild-type TrkC TM (Fig. 4d). We determined the 3.05 Å crystal structure of this chicken RPTPσ Ig1–3:TrkC LRRIg1_2Q_ complex in an attempt to visualize this additional, fourth, binding site (Table 1). Crystals grew in a new space group, P1, with three RPTPσ:TrkC complexes in the asymmetric unit (a.s.u.). These align very closely with the two complexes/a.s.u. observed in the previous P2 structure (Fig. 5b; root mean squared deviation between 446 equivalent Cα residues of the P2 complex1 relative to P2 complex2 and P1 complexes1-3 was calculated to be 0.82 Å, 0.83 Å, 0.80 Å and 0.70 Å, respectively), providing additional support for the relevance of the observed RPTPσ:TrkC interaction mode. The site 4 interface is not well resolved in this 3.05-Å resolution structure, although we do observe an additional TrkC helix (formed by residues 69–80), which interacts with RPTPσ Ig2 predominantly via potential packing of the I73 side chain against a hydrophobic region consisting of RPTPσ residues V144, Y223 and TrkC L56, T74 and L101 (Fig. 5c,d). The RPTPσ Ig2–Ig3 linker (V226-A230) and the Ig3 domain also lack well-ordered electron density in this structure. However, a R227A+R228A double mutation reduced RPTPσ:TrkC binding (Fig. 4c; Supplementary Fig. 8e,f; Supplementary Table 1), as did insertion of the meB mini-exon between R225 and V226 in SPR-binding assays, supporting the involvement of the RPTPσ Ig2–Ig3 linker in the auxilary binding site 4 (Supplementary Fig. 8i; Supplementary Table 1).

### HSPGs compete with TrkC for RPTPσ binding

**Figure 6:**
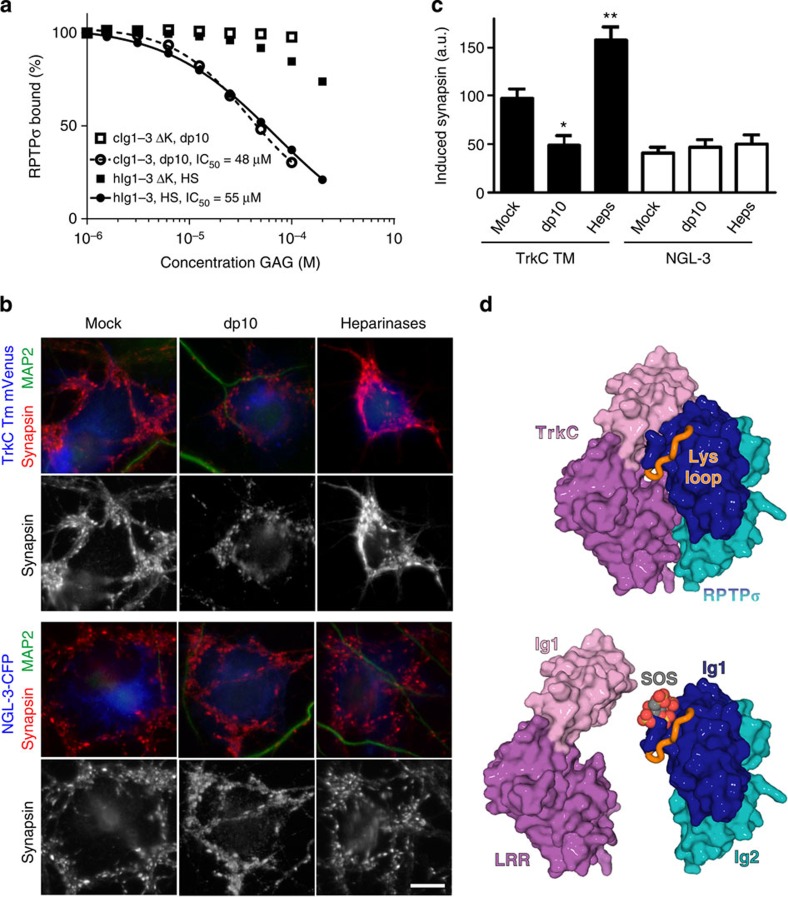
GAG-mediated inhibition of synaptic RPTPσ:TrkC interaction and function. (**a**) SPR analysis of human RPTPσ Ig1–3 and RPTPσ Ig1–3 ΔK binding to immobilized mouse TrkC LRRIg1 in the presence of increasing concentrations of HS or heparin-dp10. For sensograms see Supplementary Fig. 10a,b. (**b**,**c**) Induced synapsin clustering in rat hippocampal neurons by COS-7 cells expressing TrkC TM or NGL-3 in the presence of heparin-dp10, heparinases (Heps) or mock control. Analysis of variance *P*<0.0001, *P<0.01 and **P<0.001 compared with TrkC TM mock, whereas NGL-3 heparin-dp10 or heparainase groups were not significantly different from NGL-3 mock by *post hoc* Bonferroni’s multiple comparison test, *n*=16–26 cells from two experiments. Scale bar, 10 μM. (**d**) Illustration of the partial overlap between GAG- and TrkC-binding sites on RPTPσ. Top panel: the RPTPσ:TrkC complex, rotated 180° around the y axis relative to Fig. 2b. Lower panel: sucrose octasulphate (SOS, grey/red) is modelled in the RPTPσ GAG-binding site, an equivalent location to that observed in the LAR:SOS co-crystal structure (which is homologous with RPTPσ, PDB ID: 2YD8). SOS (or GAG) binding can out-compete the TrkC interaction with RPTPσ.

The overlap of the TrkC- and GAG-binding sites on RPTPσ, prompted us to investigate the notion that proteoglycan competition with TrkC has the potential to modulate RPTPσ function in synaptogenesis. Initially, we tested whether soluble HS or the HS-mimetic heparin-dp10 could inhibit the interactions between TrkC and either wild-type RPTPσ or a quadruple K67A+K68A+K70A+K71A mutant (RPTPσ Ig1–3 ΔK), which precludes GAG binding^14^ while retaining wild-type TrkC binding (Fig. 6; Supplementary Fig. 10a,b; Supplementary Table 1). Increasing concentrations of HS or heparin-dp10 were able to inhibit the binding of RPTPσ Ig1–3 WT, but not RPTPσ Ig1–3 ΔK to immobilized TrkC LRRIg1 (Fig. 6a; Supplementary Fig. 10a,b; Supplementary Table 1). To investigate the TrkC versus GAG competition in a cellular setting, we added heparin-dp10 to co-cultures of TrkC TM expressing COS-7 cells and rat hippocampal neurons. Induced presynaptic differentiation in the neurons decreased by twofold compared with mock-treated co-cultures upon heparin-dp10 addition (Fig. 6b,c). Furthermore, treatment of co-cultures with a mixture of heparinases I, II and III, to digest heparan sulphate GAGs, significantly enhanced presynaptic induction by TrkC TM (Fig. 6b,c), suggesting that native HSPGs may limit synapse development through RPTPσ:TrkC, by direct competition for binding (Fig. 6d). In contrast to TrkC, the interaction of RPTPσ with another trans-synaptic protein ligand, NGL-3, reported to bind at the FN1-2 domains, appears insensitive to proteoglycans. Neither heparin-dp10 nor heparinase treatment affected NGL-3-induced synaptogenesis in the co-culture system (Fig. 6b,c), and RPTPσ:NGL-3 binding in SPR assays showed no major reduction upon HS addition (Supplementary Fig. 10c,d; Supplementary Table 1).

## Discussion

**Figure 7:**
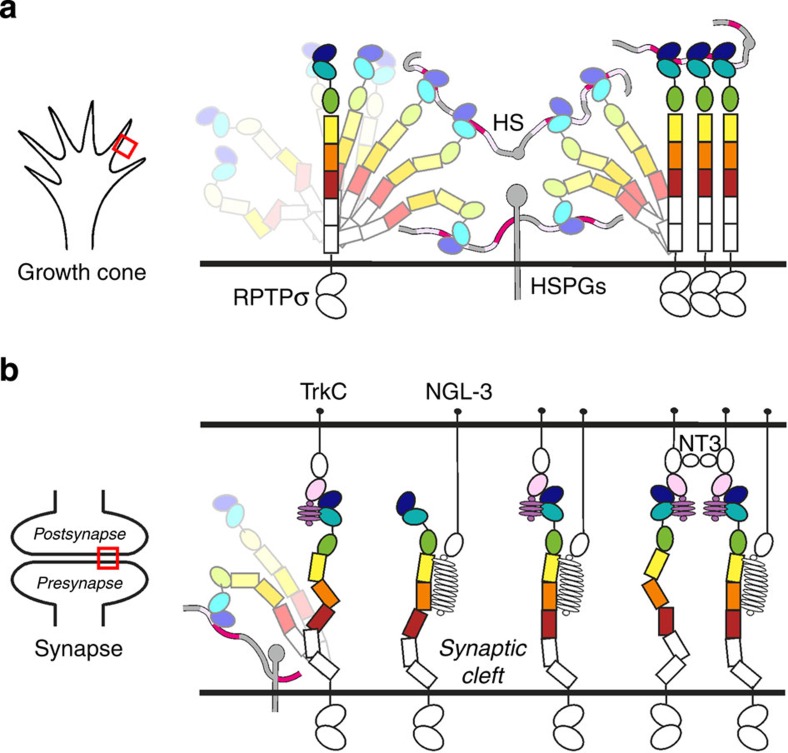
Model illustrating flexible RPTPσ ectodomain sampling of extracellular ligands. (**a**) At the growth cone, RPTPσ interacts with cell surface and basal membrane proteoglycans to mediate axonal extension. (**b**) Upon contact with target cells, to shift to the role of synaptic organizer, RPTPσ adopts elongated conformations to protrude from the presynaptic proteoglycan haze and bind postsynaptic ligands such as TrkC and NGL-3. Subsequent independent or coordinated interactions with additional synaptic ligands are shown. Red boxes (left hand panels) indicate growth cone (**a**) or synapse (**b**) regions that are illustrated in the right-hand cartoons.

While all three vertebrate type IIa RPTP family members bind NGL-3 (refs 18, 27) and interleukin-1 receptor accessory protein^19^, and both RPTPσ and RPTPδ bind to Slitrk1 and Slitrk2 (refs 20, 28), the IL-1 receptor accessory protein-like 1 (IL1RAPL1) interacts predominantly with RPTPδ^29^,^30^ and the receptor protein tyrosine kinase TrkC interacts with RPTPσ^17^. We therefore used this latter protein–protein interaction as our exemplar for trans-synaptic RPTPσ action. RPTPσ and TrkC exhibit broad and overlapping expression patterns in the adult nervous system^18^,^21^,^31^,^32^,^33^. Multiple ligand interactions and signalling pathways are disrupted in RPTPσ- and TrkC-deficient mice though, making assessment of any overlap in phenotypes difficult^34^,^35^,^36^,^37^. A direct comparison of the effect of either TrkC knockdown^17^ or RPTPσ knockout^38^,^39^ upon synapse structure and number *in vivo*, is similarly complicated by the parallel role of RPTPσ in regulating axon sprouting. The detailed explanation of RPTPσ:TrkC specificity that we offer in this study provides the information to enable the design of new experiments to dissect the precise contribution of this interaction to synaptogenesis *in vivo*. The locations of RPTPσ and TrkC are primarily reported as pre- and postsynaptic, respectively^8^,^9^, and we depict them as such in our model (Fig. 7). However, there is evidence that this may be too simplistic, for example, the type IIa RPTPs have also been reported in postsynaptic compartments^7^. The extent of flexibility we observe for the RPTPσ ectodomain, suggests that the RPTPσ:TrkC binding mode revealed by our crystal structures may also mediate *cis* interactions in the event of co-localization of the two receptors at the same cell surface.

Our data reveal several key properties that fit RPTPσ for its dual role as a synaptic signalling hub and a promoter of neuronal growth. During axonal extension, RPTPσ interacts with proteoglycan molecules through its N-terminal Ig1 domain, the clustering properties of HSPGs promoting growth cone motility^14^. The RPTPσ ectodomain architecture described here indicates that a range of conformations can be explored, permissive of *cis* interactions at the axonal surface and *trans* interactions to the general extracellular milieu including the basement membrane (Fig. 7a). Indeed, the length of the RPTPσ ectodomain may be important to extend the HSPG-binding site beyond a saturating layer of *cis* interactions at the same cell surface, similar to the sialic acid-binding Siglec family of cell surface receptors where a lengthy ectodomain is required to escape the inhibitory glycocalyx^40^. At the transition from extension to synaptogenesis, the postsynaptic neuronal surface presents an array of additional RPTPσ ligands (Fig. 7b)^8^,^9^. Synapse formation and development requires the selection of an appropriate subset of binding partners. This involves a simple kinetic competition for binding, governed by inter-molecular affinity and interaction site accessibility. Our analyses suggest that this selection may be choreographed by the interplay of binding site location and conformational flexibility in the RPTPσ ectodomain. Structural comparison of the RPTPσ:TrkC complex with our previously reported interaction mode for type IIa RPTP:GAGs^14^ shows partially overlapping binding sites (Fig. 6d). Thus, during synapse formation, and the shift from axonal growth to synaptic stability, postsynaptic TrkC must out-compete proteoglycans for RPTPσ binding, simultaneously providing an adhesive *trans* interaction and extinguishing the RPTPσ-clustering activity of HSPGs (Fig. 7b). Neuronal and glia-released proteoglycans continue to play important roles at synapses^41^,^42^, however, their contribution may involve other trans-synaptic interactions, for instance other type IIa RPTP family members or LRRTM4 (refs 43, 44).

Alternative splicing has been shown previously to control the specificity of trans-synaptic interactions of the type IIa RPTPs^8^,^17^,^20^,^28^,^29^. The impact of alternative splicing at RPTPσ mini-exon site meB on TrkC binding affinity provides a potential rheostat by which to fine-tune the balance between competing RPTPσ ligands, an analogous feature to neurexin splice site 4 control of ligand interactions at the synaptic cleft^8^,^45^,^46^. The meA mini-exon is absent from all the RPTPσ constructs used in this study, but its insertion into the Ig2 βD–βE loop (β11–β12 loop in Supplementary Fig. 1) is unlikely to affect binding of RPTPσ to TrkC, as it would lie on the opposite face to the TrkC-binding interface. Both meA and meB sites are remote from the GAG-binding surface on RPTPσ Ig1 (ref. 14) and therefore neither insertion is expected to modulate binding of RPTPσ to proteoglycans.

Trans-synaptic binding to TrkC will limit the conformational freedom of the RPTPσ ectodomain. This reduces the entropic penalty for binding at other sites on RPTPσ, an effect which can potentiate the formation of cell surface assemblies involving multiple receptor interactions^47^. For RPTPσ function at the synapse this may facilitate cooperative binding of TrkC and NGL-3, as these two binding sites are separate^18^ (Fig. 7b). TrkC can also bind the NT-3 neurotrophin, a modulator of synaptic transmission, at the second Ig domain^48^ (Fig. 7b), which adds a further, distinctive stoichiometry to this network of trans-synaptic interactions, by specifically triggering dimerization of TrkC and hence RPTPσ. Other soluble modulators, such as the astrocyte-derived HSPGs glypican-4 and glypican-6, may provide an alternative strategy to regulate this system^41^. The stoichiometry and architecture of higher-order trans-synaptic complexes, and their impact on RPTPσ enzymatic activity, remain important questions to address in the future. Formally, we cannot yet exclude a scenario where the simple kinetics of competition between ligands may be sufficient to explain the phenotypes observed in our cellular assays. However, given the geometrical constraints within which the RPTPσ ectodomain has to operate at points of cellular contact, with ligands present in both *cis* and *trans* orientations, it is very likely that the RPTPσ ectodomain flexibility is required in a physiological context.

A series of proteolytic processing events have been reported for the type IIa RPTPs at the cell surface, involving initial shedding of the ectodomain, prior to release of the intracellular catalytic domains^49^. In our crystallization trials, we have potentially identified a further receptor-cleavage site at the consensus furin-like protease motif RVRR on the Ig2–Ig3 linker, which would be disrupted in RPTPσ isoforms containing mini-exon meB. Cleavage of either RPTPσ Ig1–2 or ectodomain fragments would first decouple the receptor phosphatase activity from regulation via both proteoglycan and TrkC binding, and second the released soluble RPTPσ fragments would be able to compete with the remaining intact receptors for binding to these same ligands. Knockout of the *Drosophila* type IIa RPTP dLAR, can be rescued by reintroduction of a catalytically inactive receptor, but not by a dLAR construct lacking the second inactive phosphatase domain (D2)^50^. It remains to be determined whether RPTPσ ectodomain–ligand interactions may regulate the availability of RPTPσ D2 for binding downstream intracellular partners^8^,^9^, but any such mode of regulation would also be ablated by extracellular RPTPσ cleavage events.

In conclusion, our results suggest how the RPTPσ nexus utilizes a series of filtering mechanisms to discriminate between binding options, and ultimately integrate the signalling inputs essential for the transition from neuronal growth to synapse organization. Rigidity has been demonstrated to be central for the comparatively simpler adhesion molecule function^24^,^25^,^26^. In contrast, the properties of RPTPσ described here, prompt the notion of how ectodomain flexibility can allow a cell surface receptor to integrate a broad spectrum of ligand interactions into distinct functional outcomes.

## Methods

### Construct design and cloning

Human RPTPσ Ig1–3, Ig1-FN3 and sEcto pHLsec constructs were reported previously^14^. The chick RPTPσ Ig1–3 construct (amino acids 29–316, NCBI Ref. Seq. NM_205407.1) was cloned into pHLsec and pHL-Avitag3 vectors^51^. A series of chick RPTPσ Ig1–3 mutant constructs were generated by PCR: K67A+K68A+K70A+K71A (chick RPTPσ Ig1–3 ΔK), N73S+S74N, R96A+R99A, P97V+T98H, K203A, Y223S and R227A+R228A and subsequently cloned into the pHL-Avitag3 vector. Human RPTPσ Ig1–3 K68A+K69A+K71A+K72A (human RPTPσ Ig1–3 ΔK), R97A+R100A, Y224S and R228A+R229A C-terminal Avitag constructs were similarly constructed. Human RPTPδ sEcto (amino acids 21–833, NCBI Ref. Seq. BC106713.1), and human RPTP LAR Ecto (amino acids 30–1,260, NCBI Ref. Seq. NM_002840.3) were also cloned into the pHL-Avitag3 vector.

A synthetic clone for chick TrkC was commercially synthesized (Source Bioscience); to include amino-acid residues 32–302 (NCBI Ref. Seq. NM_205169.1), but lack residues 63–77 and include the following point mutations: N163Q, N232Q, N259Q, N267Q and N294Q, to reduce the number of *N*-linked glycosylation sites. This construct (chick TrkC LRRIg1_cryst_) was cloned into the pHL-Avitag3 vector. A series of further chick TrkC LRRIg1 mutant constructs were generated by PCR: D240A+D242A, re-addition of residues 63–77 (chick TrkC LRRIg1_2N_) and re-addition of residues 63–77 with N68Q+N72Q point mutations (chick TrkC LRRIg1_2Q_).

A series of mouse TrkC constructs (NCBI Ref. Seq. BC139764.1) were cloned into both pHLsec and pHL-Avitag3 vectors: LRR (amino acids 32–208), LRRIg1 (amino acids 32–302) and LRRIg2 (amino acids 32–398). A mouse TrkC TM (amino acids 32–463) construct was cloned into the pHLsec-monoVenus vector. The following mouse TrkC mutant constructs were generated by PCR: D240A+D242A (LRRIg1 and TM constructs), N68Q+N72Q (LRRIg1_2Q_ and TM_2Q_) and removal of residues 63–77 (LRRIg1_cryst_). Mouse NGL-3 (NCBI Ref. Seq. BC060263.1) ectodomain (NGL-3 Ecto; amino acids 1–574) and full-length (NGL-3 FL; amino acids 1–709) constructs were cloned into pHL-Avitag3 and pHLsec-monoCerulean vectors, respectively.

### Protein purification and crystallization

For crystallization purposes, constructs were expressed in either HEK-293S GnTI^−^ cells (chicken RPTPσ Ig1–3, TrkC LRRIg1_cryst_ and TrkC LRRIg1_2Q_) or HEK293-T cells treated with kifunensine (human RPTPσ Ig1-FN3) following transient transfection using polyethylenimine^51^. The proteins were purified from 0.2-μm-filtered cell culture media by immobilized nickel affinity chromatography (Chelating Sepharose Fast Flow, GE Healthcare) followed by size-exclusion chromatography in 10 mM HEPES, 150 mM NaCl, pH 7.5.

Crystallization trials, using 100 nl protein solution plus 100 nl reservoir solution in sitting-drop vapour diffusion format were set up in 96-well Greiner plates using a Cartesian Technologies robot, and plates were subsequently maintained at 20.5 °C in a TAP Homebase storage vault. The crystallization conditions yielding diffraction quality crystals were: 10% polyethylene glycol (PEG) 20 k, 20% PEG MME 550, 0.1 M bicine/Tris pH 8.5, 0.03 M sodium nitrate, 0.03 M disodium hydrogen phosphate, 0.03 M ammonium sulphate (chicken RPTPσ Ig1–2:TrkC LRRIg1_cryst_ complex 2.5 Å data set), 10% w/v PEG MME 5 k, 0.1 M HEPES pH 7.0, 5% w/v Tacsimate (chicken RPTPσ Ig1–3:TrkC LRRIg1_2Q_ complex 3.05 Å data set) and 10% PEG 400, 0.01 M magnesium chloride, 0.1 M potassium chloride, 0.05 M MES pH 6.0 (human RPTPσ Ig1-FN3).

### Data collection and processing

Crystals were cryoprotected using a 25–30% solution of ethylene glycol (chicken RPTPσ Ig1–2:TrkC LRRIg1_cryst_ and RPTPσ Ig1–3:TrkC LRRIg1_2Q_ complexes) or 25% propylene glycol (human RPTPσ Ig1-FN3) and then flash-cooled at 100 K. X-ray diffraction data were collected at the I03 (chicken RPTPσ Ig1–3:TrkC LRRIg1_2Q_; wavelength 0.9763 Å) and I04 (chicken RPTPσ Ig1–2:TrkC LRRIg1_cryst_; wavelength 0.9200 Å) beamlines Diamond Light Source, Oxfordshire, UK and the ID29 (human RPTPσ Ig1-FN3; wavelength 0.9788 Å) beamline at the European Synchotron Radiation Facility, Grenoble, France. The diffraction images were indexed, integrated, scaled and merged using the xia2 data-processing suite^52^. We used *R*_pim_, *I*/*σI* and CC_1/2_ (ref. 53) statistics in the highest-resolution shell (with criteria *R*_pim_<100%, *I*/*σI*>1.5 and CC_1/2_>50%) to determine our high-resolution cutoffs (see Table 1).

Molecular replacement was used to phase all three crystal structures, using human and chicken RPTPσ Ig1–2 (PDB ID: 2YD3 and 2YD4), human RPTPσ Ig3 (from PDB ID: 2YD9), a Cα model for human RPTP LAR FN4 (PDB ID: 2DJU) and models of the chicken TrkC LRR and first Ig domain (Ig1) generated using the SWISS-MODEL web interface^54^, as search models in Phaser^55^. Manual model adjustment was performed in Coot^56^ and the Refmac^57^, Phenix^58^ and Buster (Global Phasing Ltd)^59^ suites used for refinement (applying translation libration screw-motion restraints for all structures and local non-crystallographic symmetry restraints for RPTPσ:TrkC complex structures). Stereochemical properties of all models were accessed using MolProbity^60^. Ramachandran statistics: human RPTPσ Ig1-FN3, 94.0% most favoured, 6.0% additionally allowed and no disallowed rotamers; chicken RPTPσ Ig1–2:TrkC LRRIg1_cryst_, 98% most favoured, 2% additionally allowed and no disallowed rotamers; chicken RPTPσ Ig1–3:TrkC LRRIg1_2Q_, 96.6% most favoured, 3.2% additionally allowed and 0.2% disallowed rotamers. Full data collection and refinement statistics are given in Table 1.

The 3.15-Å human RPTPσ Ig1-FN3 crystal structure contains one molecule per asymmetric unit; amino residues 35–601, an additional C-terminal G residue derived from the expression vector and two GlcNAc residues at *N*-linked glycosylation sites N250 and N295.

The 2.5 Å chicken RPTPσ Ig1–2:TrkC LRRIg1_cryst_ crystal structure contains amino-acid residues 32–302 for two molecules, A and B, of TrkC LRRIg1 (lacking residues 63–77 absent in the TrkC LRRIg1_cryst_ expression construct and residues 258–261 on a disordered Ig1 loop, but with ETG (A) and G (B) additional N-term residues and GT (A) and G (B) additional C-term residues derived from the expression plasmid) and residues 29–227, 29–226 and 29–228 for three molecules, C, D and E, of RPTPσ Ig1–2 (with the exception of a disordered loop for each molecule, 68–71(C), 68–70(D) and 68–73(E)). Five TrkC *N*-linked glycosylation sites were modelled; initial *N*-acetylglucosamine (GlcNAc) residues, covalently bonded to N79 (A and B), N203 (A) and N272 (A and B) were included in the structure. Inspection of the crystal packing clearly demonstrates that the Ig3 domains of the RPTPσ Ig1–3 crystallization protein are not present in these crystals. Proteolytic cleavage is the most likely reason for their absence, as we have commonly seen cleavage of wild-type RPTPσ Ig1–3 at the Ig2–Ig3 linker during crystallization trials. The previously reported human RPTPσ Ig1–2 crystal structure was obtained via proteolysis of Ig1–3 during crystallization (PDB ID 2YD3). To produce crystals of intact human RPTPσ Ig1–3, R227Q+R228N (residue numbering relative to chicken RPTPσ) point mutations were previously introduced to disrupt a potential furin protease motif^14^. However, RPTPσ R227 and R228 are required for full RPTPσ:TrkC binding (Fig. 4c; Supplementary Fig. 8e,f; Supplementary Table 1) and therefore the RPTPσ Ig1–3 R227Q+R228N protein was not used in crystallization trials in this study.

The 3.05 Å chicken RPTPσ Ig1–3:TrkC LRRIg1_2Q_ crystal structure contains amino-acid residues 32–302 for three molecules, A, B and C, of TrkC LRRIg1 (lacking residues 59–68 (A), 59–68 (B) and 57–73 (C), but with TG (A), G (B) and G (C) additional N-term residues derived from the expression plasmid) and residues 29–226, 29–227 and 29–225 for three molecules, D, E and F, of RPTPσ Ig1–3 (with the exception of residues 67–71 in the disordered ‘Lys-loop’ for each molecule, for which density was visible, but a single conformation could not be built). Electron density was visible to indicate the presence of *N*-linked glycosylation adjacent to N79 in TrkC molecules A–C, but only in molecule A could an initial GlcNAc residue be successfully refined. There was also evidence that N218 (electron density corresponding to molecule B) and N272 (electron density corresponding to molecule A) are also *N*-linked glycosylation sites. Sparse electron density is visible for RPTPσ Ig3, suggesting disorder/multiple conformations of this domain.

The assignment of secondary-structure elements was performed using ksdssp^61^. The superposition of atomic models to compare the domain architecture between different structures was performed using SHP^62^, based on Cα positions. Crystallographic figures were created using PyMOL (Schrödinger, LLC) and APBS^63^ was used to calculate the electrostatic potential of solvent-accessible surfaces.

### Surface Plasmon Resonance

All SPR experiments were performed on BIAcore T100 or T200 instruments. Ligand constructs were expressed in HEK293-T cells with no kifunensine treatment and purified via Ni-affinity chromatography. All contained a C-terminal BirA recognition site (Avitag) and were biotinylated enzymatically prior to immobilization to the surface of CM5 sensor chips (BIAcore) pre-coated with streptavidin (Sigma) using the BIAcore amine-coupling kit. Analyte constructs were expressed in either GnTI^−^ HEK293-S or HEK293-T cells and purified as for crystallization purposes, described above.

Unless otherwise stated, experiments were all performed in 10 mM HEPES pH 7.5, 150 mM NaCl, 0.01% Tween 20, at a temperature of 25 °C and a flowrate of 20 μl min^−1^. Typically, each analyte sample was injected across the chip surfaces for 120 s and then a 300s dissociation time was included to allow the signal to return to baseline. For equilibrium SPR experiments, serial two- or threefold dilutions of protein analyte were sequentially injected and all injection series were repeated in duplicate. No regeneration of the chip surfaces was required between analyte injections, except when measuring the interaction between chicken TrkC LRRIg1_2Q_ and chicken RPTPσ Ig1–3, when a 30s injection of 1 M MgCl_2_ was sufficient for the signal to return to baseline.

Scrubber2 (BioLogic Software) and Prism (GraphPad Software) were used for data analysis assuming the Langmuir model and a 1:1 ligand to analyte ratio. The signal for experimental flow cells was corrected by initial subtraction of a blank (only buffer injected as analyte across the flow cell of interest), followed by the subtraction of the reference signal from a mock-coupled flow cell (streptavidin, but no ligand bound). To estimate half-maximal inhibitory concentration values for heparan sulphate or heparin-dp10 inhibition of RPTPσ:TrkC binding, nonlinear regression in Prism (GraphPad Software) was used to fit a variable-slope dose–response curve to the experimental data, fixing top and bottom values according to control measurements (top; response units when no inhibitor present and bottom; response units when no protein analyte or inhibitor present), while the Hill Slope coefficient and half-maximal inhibitory concentration variables were left unrestrained.

### Multi-Angle Light Scattering

MALS experiments were carried out on a Wyatt MALS/AFFFF System (Wyatt Technologies). Human RPTPσ Ig1-FN3 and mouse TrkC LRRIg1 proteins were expressed in GnTI^−^ HEK-293S cells and purified as described above. Size-exclusion chromatography was performed in 10 mM Tris, 50 mM NaCl, pH 7.5 on a Superdex200 HR10/30 column (GE Healthcare), attached to an Agilent chromatography system. An Optilab rEX Refractive Index detector and a Dawn Helios II Multi-Angle Light Scattering detector recorded the refractive index and light scattering of the samples upon elution from the size-exclusion column. The Wyatt software ASTRA was used to analyse all the data collected.

### Negative-stain EM

Human RPTPσ Ig1-FN3 and sEcto proteins were negatively stained with 0.7% uranyl formate^64^. Images were recorded using an FEI electron microscope equipped with a LaB_6_ filament operated at an acceleration voltage of 200 keV at a magnification of × 55,000 and a defocus value of approximately −1.5 μm. All images were recorded using SO-163 film and developed with a Kodak D-19 developer at full strength for 12 min at 20 °C. The electron micrographs were digitized with a CoolScan 9000 (Nikon) using a step size 6.35 μm, and the pixels were binned by a factor of 3. As a result, the specimen level pixel size was at 3.8 Å. To generate projection averages, particles were interactively selected using the WEB display program in SPIDER^65^ and windowed into 90 × 90-pixel (human RPTPσ Ig1-FN3) and 100 × 100-pixel (human RPTPσ sEcto) images. Class averages were calculated using these windowed images over 10 cycles of K-means classification and multi-reference alignment specifying 150 classes^64^.

### SAXS

RPTPσ Ig1-FN3 and sEcto proteins were deglycosylated by Endo-F1 and purified by SEC immediately prior to data collection. Solution scattering data were collected at beamline BM29 of the European Synchotron Radiation Facility^66^ at 293 K within a momentum transfer range of 0.01 Å^−1^<*q*<0.45 Å^−1^, where *q*=4*π*sin(*θ*)/*λ* and 2*θ* is the scattering angle. X-ray wavelength was 0.995 Å and data were collected on a Pilatus 1M detector. RPTPσ Ig1-FN3 was measured at 1.33 and 5.33 g l^−1^ and RPTPσ sEcto at 1.00 and 4.00 g l^−1^. Data reduction and calculation of invariants was carried out using standard protocols implemented in the ATSAS software suite^67^. A merged data set was obtained by merging the low-angle part of the low-concentration data set with the high-angle part of the high-concentration data set.

A pool of 10,000 independent model conformers was constructed using the program RANCH^67^ for both RPTPσ Ig1-FN3 and sEcto by treating individual domains as beads-on-a-string. For RPTPσ sEcto, a homology model for domains 8 and 9 was created using SWISS-MODEL^54^. Both pools contained conformer shapes ranging from collapsed or U-shaped to fully extended, as evidenced by their Gaussian *R*_G_ and *D*_MAX_ distributions. Ensemble selection using the experimental SAXS data as constraint with the programs GAJOE^67^ or MES^68^ indicated predominantly extended conformers, consistent with the experimentally determined *R*_G_ and *D*_MAX_. The MES-selected models were used as starting structures for further modelling. Missing loops and N- and C termini were added in extended conformations using the program Modeller. All-atom simulations of RPTPσ Ig1-FN3 and sEcto was performed using the program AllosMod. For each starting structure, 30 independent pools of 100 models were generated. For the combined pool, calculation and fitting of scattering patterns was performed using the program FoXS, and automated selection of the minimal set of models satisfying the scattering data was performed using the program MES; this whole procedure was automated using the AllosMod-FoXS web server^68^.

### Neuron-COS cell co-culture assays

Dissociated primary hippocampal neuron cultures were prepared from embryonic day 18 rat embryos^69^. For co-culture assays, COS-7 cells were transfected and 24 h later were seeded onto neurons at 14 days *in vitro*^70^. As indicated, co-culture coverslips were incubated with 0.2 U ml^−1^ heparinase I, II and III or with 30 μg ml^−1^ (~10 μM) heparin-dp10 (Iduron Ltd, UK) in glial conditioned medium. Co-cultures were fixed with 4% formaldehyde and 4% sucrose in phosphate-buffered saline (PBS) (pH 7.4) followed by permeabilization with 0.2% Triton X-100. For cell surface staining of TrkC, COS-7 cells were fixed without permeabilization. Fixed cultures were blocked in 3% bovine serum albumin and 5% normal goat serum in PBS for 30 min at 37 °C, and primary antibodies (overnight incubation at 4 °C) then secondary antibodies (1 h at 37 °C) were applied in 3% bovine serum albumin and 5% normal goat serum in PBS. Coverslips were mounted in elvanol (Tris-HCl, glycerol and polyvinyl alcohol, with 2% 1,4-diazabicyclo[2,2,2]octane). The primary antibodies were: anti-TrkC (1:500; C44H5; Cell Signaling), anti-synapsin I (rabbit, 1:2,000; Millipore; AB1543P) for presynaptic terminals, anti-MAP2 (chicken polyclonal IgY; 1:2,000; Abcam; ab5392) for dendrites and anti-dephospho-tau (mouse mIgG2a 1:2,000, clone PC1C6; Millipore; MAB3420) for axons.

All image acquisitions, analyses and quantifications were performed by investigators blind to the experimental condition. For co-cultures, fields for imaging were chosen only by the Cyan Fluorescent Protein (CFP) or mVenus and phase-contrast channels, for the presence of CFP or mVenus-positive COS-7 cells in a neurite-rich region. The synapsin channel was thresholded and the total intensity of puncta within all regions positive for both CFP or mVenus and dephospho-tau but negative for MAP2 was measured. Analysis was performed using Fiji (ImageJ2), and GraphPad Prism 5. All data are reported as mean±s.e.m.

## 

## Author contributions

A.R.A., E.Y.J. and C.H.C. designed the project and all authors contributed to data analysis and preparation of the manuscript. W.L. transiently expressed all proteins. C.H.C. and N.M. cloned and purified all constructs and performed SPR and crystallographic analyses for the RPTPσ:TrkC complexes. C.H.C. solved the RPTPσ Ig1-FN3 crystal structure. T.N. and C.H.C. performed the negative-stain electron microscopy and processed and analysed these data. N.M. and J.E. collected and analysed all SAXS data. A.W.S. carried out cellular assays. P.Z. performed COS-hippocampal cell culture experiments and analysed these data together with A.M.C.

## Additional information

**How to cite this article:** Coles, C. H. *et al.* Structural basis for extracellular *cis* and *trans* RPTPσ signal competition in synaptogenesis. *Nat. Commun.* 5:5209 doi: 10.1038/ncomms6209 (2014).

## Supplementary Material

Supplementary Information Supplementary Figures 1-10, Supplementary Table 1 and Supplementary References.

## Figures and Tables

**Table 1:** Data collection and refinement statistics.

	**hRPTPσ Ig1-FN3**	**cRPTPσ Ig1-2 +cTrkC LRRIg1**_**cryst**_	**cRPTPσ Ig1-3 +cTrkC LRRIg1**_**2Q**_
*Data collection*			
Space group	P6_1_22	P2	P1
*Cell dimensions*			
*a*, *b*, *c* (Å)	198.8, 198.8, 132.4	68.3, 122.2, 98.6	84.4, 93.1, 99.4
*α*, *β*, *γ* (°)	90.0, 90.0, 120.0	90.0, 109.8, 90.0	73.4, 89.5, 74.2
Resolution (Å)	99.40–3.15 (3.23–3.15)*	63.96–2.50 (2.56–2.50)	81.02–3.05 (3.13–3.05)
*R*_merge_	7.8 (99.1)	19.2 (142.4)	6.9 (34.7)
*R*_*pim*_†	3.2 (41.0)	5.7 (57.2)	5.6 (60.3)
CC_1/2_‡	99.8 (63.9)	99.5 (58.4)	99.8 (67.2)
*I*/*σI*	17.8 (2.1)	10.7 (1.6)	8.8 (1.5)
Completeness (%)	95.6 (96.1)	99.8 (99.3)	96.3 (96.6)
Redundancy	7.6 (7.5)	11.6 (7.1)	1.8 (1.8)
			
*Refinement*			
Resolution (Å)	99.40–3.15 (3.23–3.15)	63.96–2.50 (2.56–2.50)	81.02–3.05 (3.13–3.05)
No. reflections	25,619 (1,858)	52,652 (3,833)	51,063 (3,823)
*R*_work_/*R*_free_	23.4 (37.1)/26.5 (37.7)	20.9 (32.2)/24.7 (36.2)	22.6 (37.2)/24.0 (38.6)
			
*No. of atoms*			
Protein	4,380	8,536	10,630
NAGs	2	5	1
SO_4_^2−^ ions	—	2	—
Water	—	118	—
			
*B-factors*			
Protein	122.2	57.2	116.4
NAGs	153.0	83.0	176.3
SO_4_^2−^ ions	—	90.9	—
Water	—	45.0	—
			
*R.m.s.d.*			
Bond lengths (Å)	0.005	0.008	0.006
Bond angles (°)	1.008	1.243	1.045

RPTP, Receptor protein tyrosine phosphatase; r.m.s.d., root mean squared deviation.

^*^Values in parentheses are for highest-resolution shell. Each structure is based on a single crystal.

^†^*R*_pim_ (the precision-indicating merging *R*-value)=1/(*N*−1) × *R*_merge_, where *N* is the redundancy.

^‡^CC_1/2_ is the mean intensity correlation coefficient of half-data sets^53^.
