# Supplementary material for: Structural basis for extracellular cis and trans RPTPσ signal competition in synaptogenesis
Source: Nat Commun. 2014 Nov 11;5:5209. doi: 10.1038/ncomms6209 (PMC4239663; doi:10.1038/ncomms6209)
Supplement: Supplementary Information — Supplementary Figures 1-10, Supplementary Table 1 and Supplementary References. [file ncomms6209-s1.pdf]

Supplementary Information

for

Structural basis for extracellular *cis* and *trans* RPTPσ signal competition in synaptogenesis

Charlotte H. Coles, Nikolaos Mitakidis, Peng Zhang, Jonathan Elegheert, Weixian Lu, Andrew W. Stoker, Terunaga Nakagawa, Ann Marie Craig, E. Yvonne Jones[†], A. Radu Aricescu[†]

[†]To whom correspondence should be addressed. Email: radu@strubi.ox.ac.uk (A.R.A.); yvonne@strubi.ox.ac.uk (E.Y.J).

This pdf file includes:

Supplementary Figures 1-10

Supplementary Table 1

Additional references for Supplementary Figures

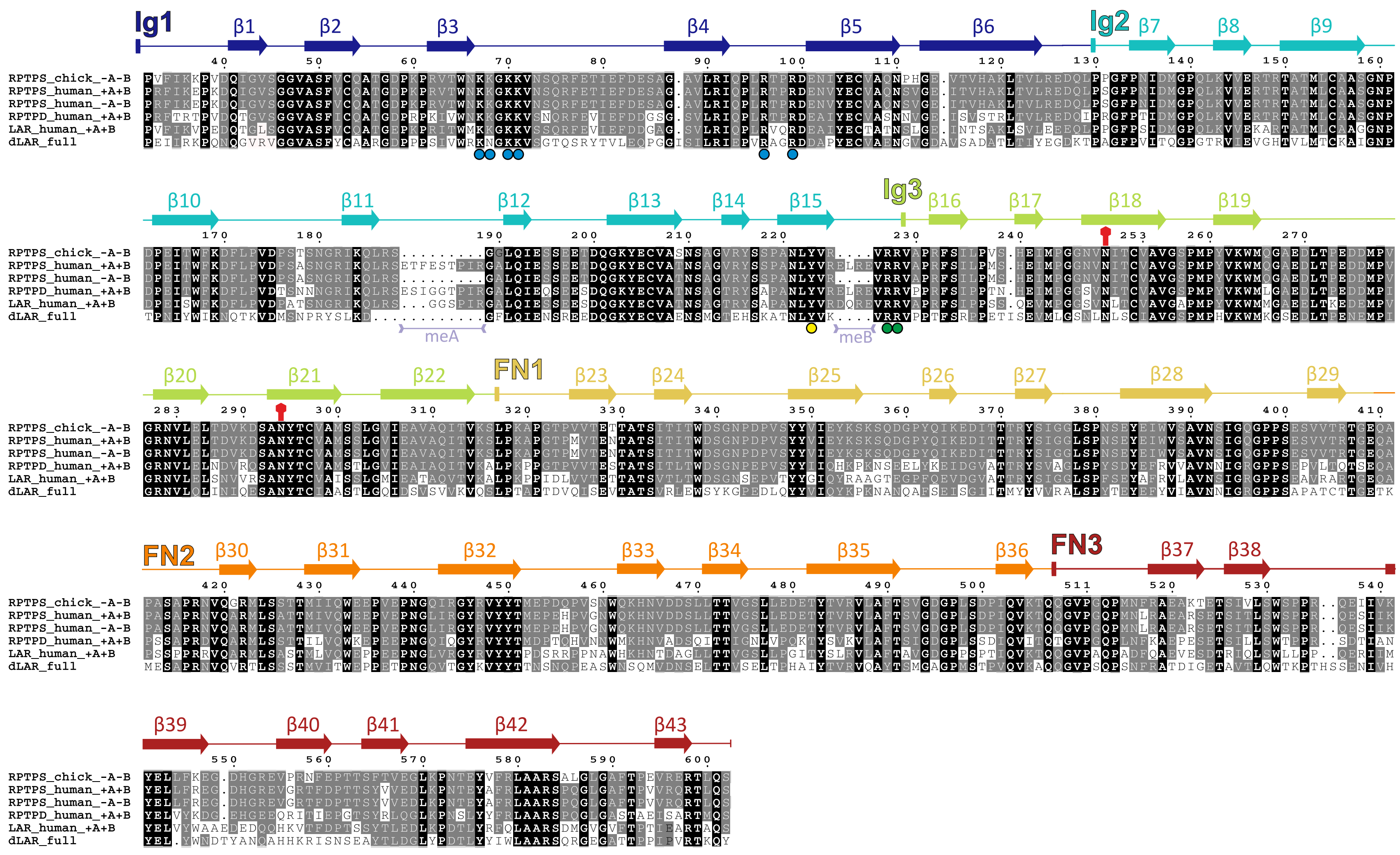

Supplementary Figure 1. Sequence alignment of the six N-terminal domains (Ig1-FN3) of representative type IIa RPTP family members. Amino acid sequences taken from the following sources: chicken RPTPσ (lacking meA and meB mini exons; NM_205407), human RPTPσ (with meA and meB; Q13332, without meA and meB Q13332-6), human RPTPδ (with meA and meB; P23468), human LAR (with meA and meB; XP_005271135) and *Drosophila* LAR (P16621). The secondary structure elements observed for the RPTPσ Ig1-FN3 crystal structure are coloured above the sequence alignment according to the schematic in Figure 1a: Ig1; blue, Ig2;cyan, Ig3; green, FN1; yellow, FN2; orange and FN3; red (assigned using ksdssp[S1]). Coloured circles below the alignment indicate amino acid residues mutated in our binding and cellular assays; K67, K68, K70, K71, R96 and R99 (binding site 1; blue), Y223 (binding site 3; yellow), R227 and R228 (binding site 4; green). Red sticks highlight N-linked glycosylation sites and residues underlined in purple correspond to meA and meB mini exons. Numbering above the alignment corresponds to the chicken RPTPσ sequence.

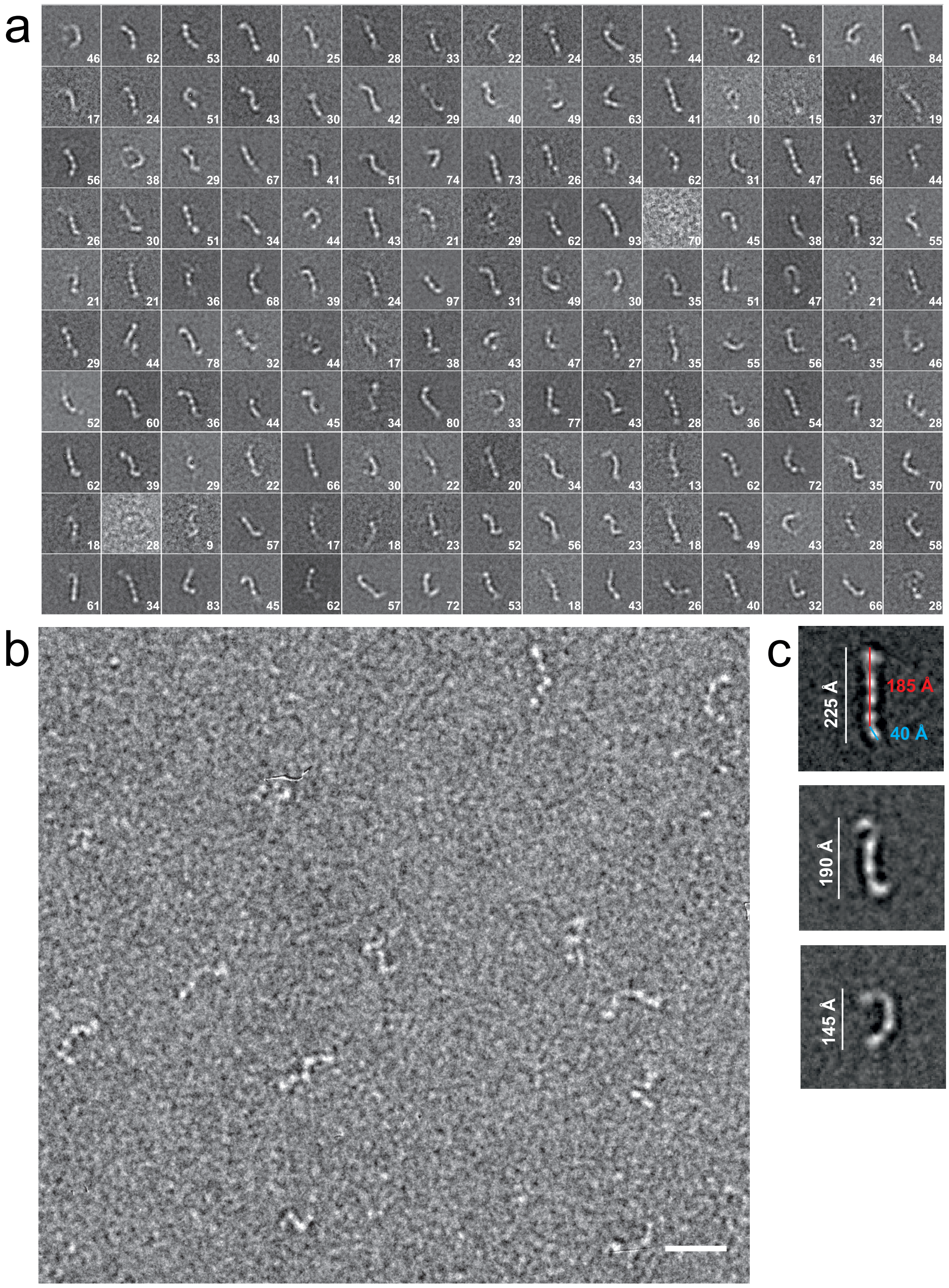

Supplementary Figure 2. Class averages of RPTPσ Ig1-FN3 particles. (**a**) 6315 particles were visually selected and classified into 150 classes by K-means classification and multi-reference alignment. The number of particles used to generate each class average is indicated in the bottom right of each image. The size of each image is 34.2 nm by 34.2 nm. (**b**) Representative area of a raw negative stain EM micrograph of RPTPσ Ig1-FN3. Scale bar; 20nm. (**c**) Illustration of how particle dimensions were estimated from representative class averages. The contribution of the protein solvation sphere and negative stain can not be accurately determined, but these estimates approximately agree with crystal structure derived lengths of Ig1-FN2 (170 Å) and FN3 (40 Å) and a height of 175 Å (comparable to the class average in the middle panel in (c)).

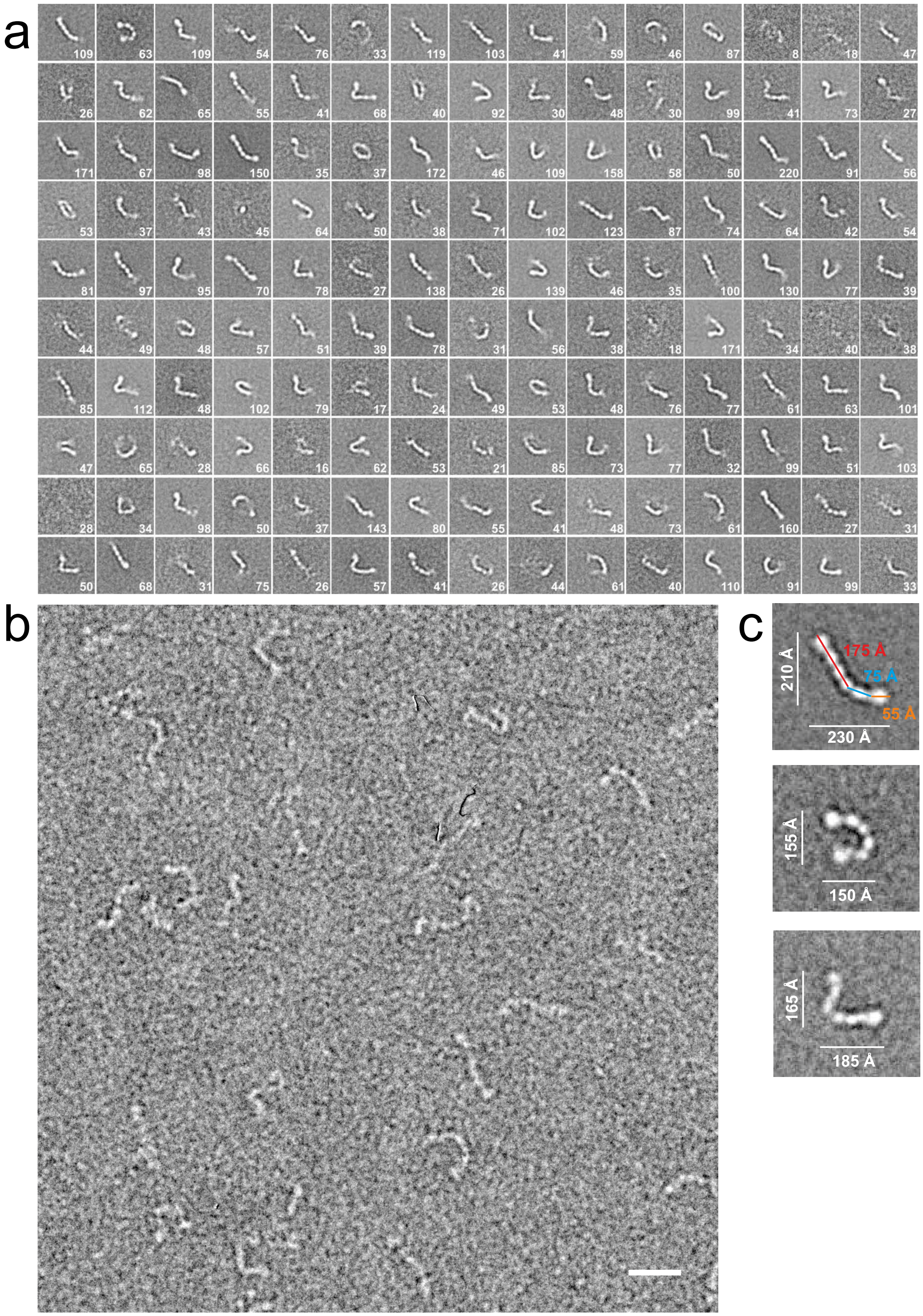

Supplementary Figure 3. Class averages of RPTPσ sEcto particles. (**a**) 9895 particles were visually selected and classified into 150 classes by K-means classification and multi-reference alignment. The number of particles used to generate each class average is indicated in the bottom right of each image. The size of each image is 38 nm by 38 nm. (**b**) Representative area of a raw negative stain EM micrograph of RPTPσ sEcto. Scale bar; 20 nm. (**c**) Illustration of how particle dimensions were estimated from representative class averages. These are approximations, as the contribution of the protein solvation sphere and negative stain can not be accurately determined, but suggest that a certain degree of flexibilty is required for the RPTPσ ectodomain to fit within the ~240 Å average width calculated for excitatory synapses[S2].

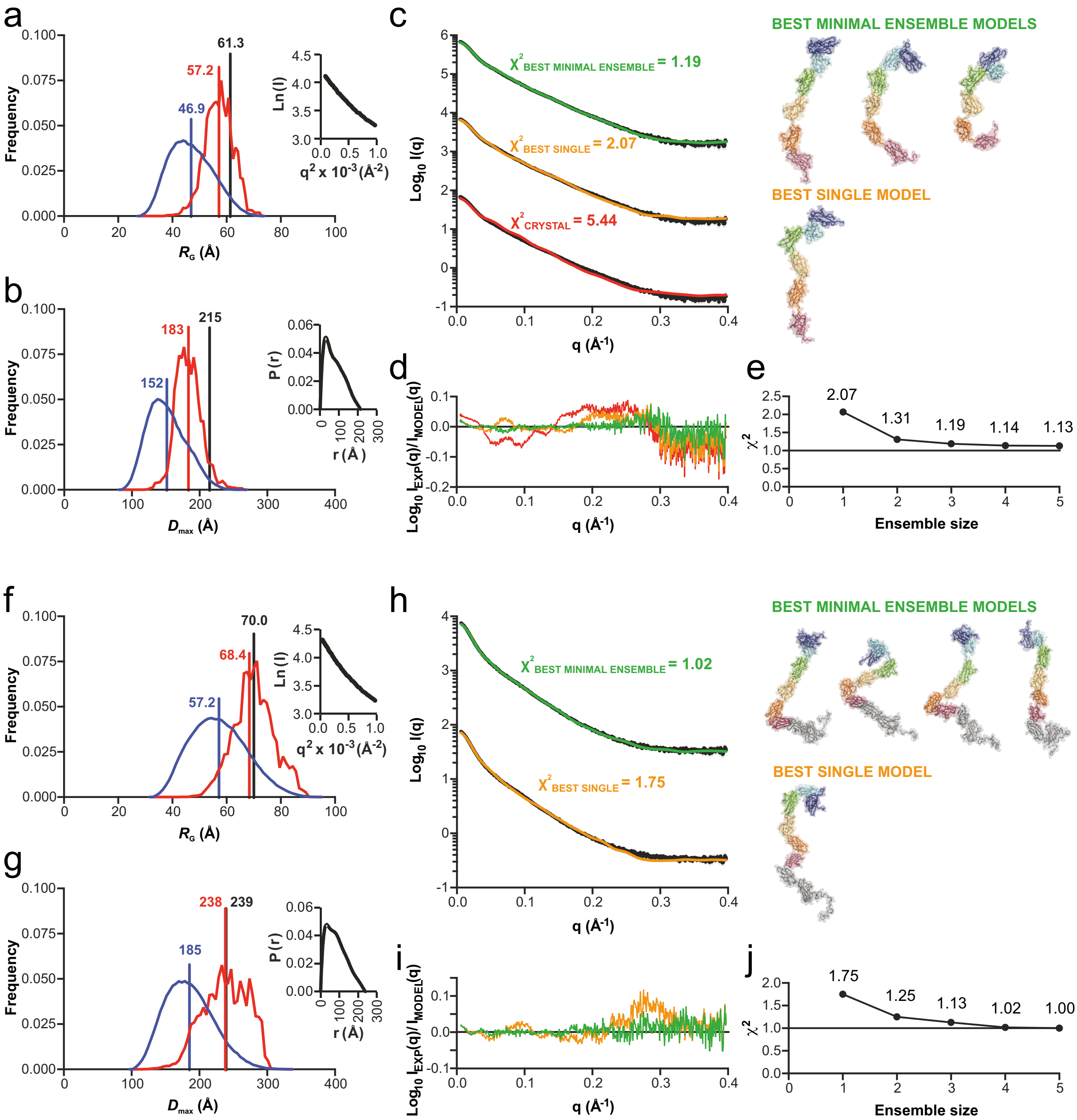

Supplementary Figure 4. Small angle X-ray scattering (SAXS) analysis of RPTPσ ectodomain conformation in solution. (**a-e**) SAXS analysis of RPTPσ Ig1-FN3. (**a**) R_G and (**b**) D_{MAX} distribution of the 10,000 models generated by RANCH (blue) and of the GAJOE-selected ensemble (red). Mean values are indicated by blue (model pool) and red (selection) lines. Mean experimental values are indicated by a black line. Insets show the experimentally determined Guinier region (a) and pair distance distribution function (b). (**c**) Solution structure of RPTPσ Ig1-FN3. Experimental scattering curves (black) and calculated scattering patterns (coloured) are shown to a maximal momentum transfer of q = 0.40 Å^{-1}. Individual data:fit pairs are displaced along an arbitrary y axis to allow for better visualization. Bottom curve: Ig1-FN3 crystal structure (red). Middle curve: best single model (orange). Top curve: best minimal model ensemble (green). Best single and minimal ensemble models are shown in ribbon and surface representation. (**d**) Fitting residuals of the experimental scattering curves and calculated scattering patterns for the Ig1-FN3 crystal structure (red), best single model (orange) and best minimal ensemble (green). (**e**) χ^2 versus ensemble size. (**f-j**) SAXS analysis of RPTPσ sEcto; equivalent to (a-e).

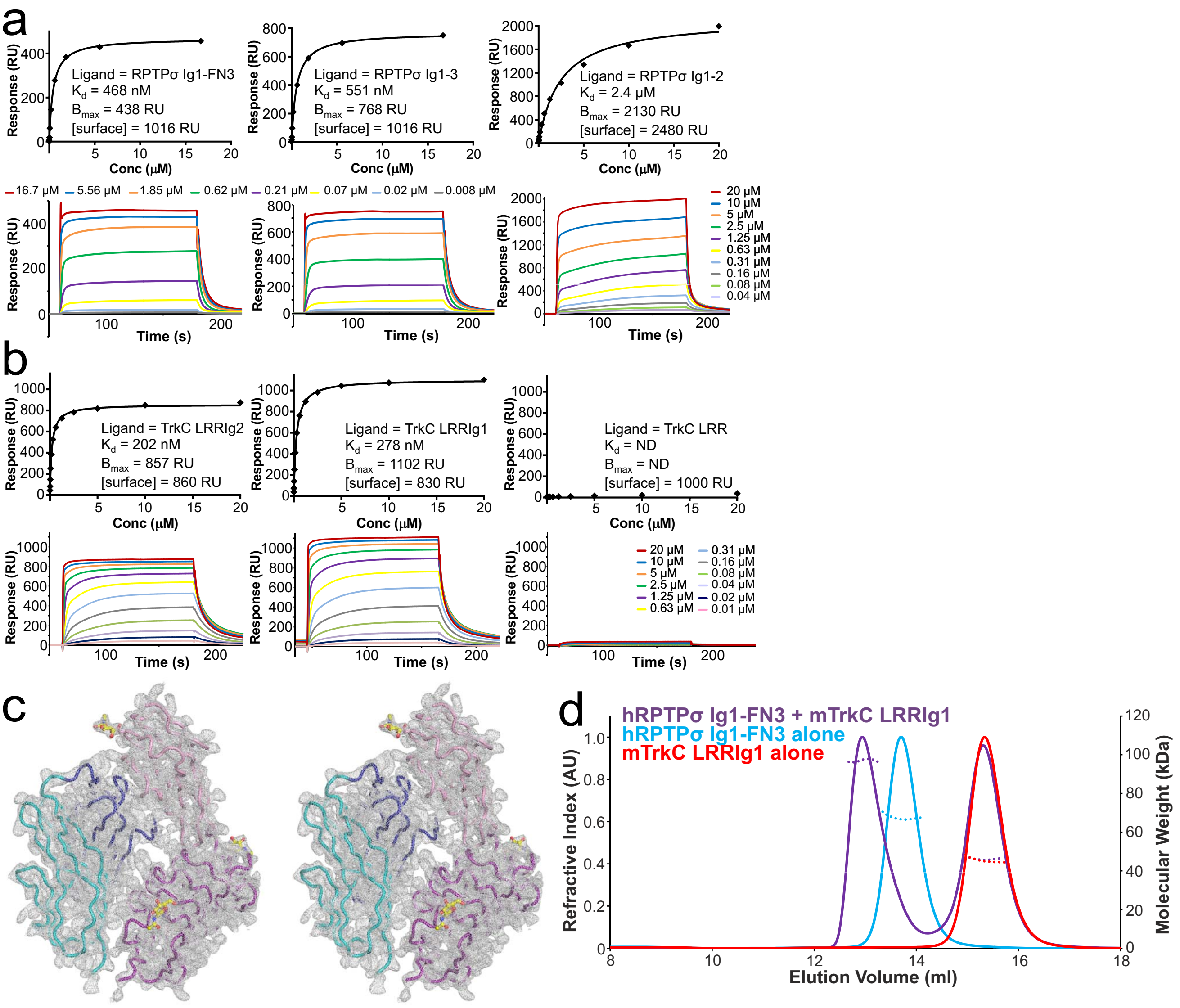

Supplementary Figure 5. Minimal domain requirements and stoichiometry of RPTPσ-TrkC interaction. (**a**) SPR analysis of mouse TrkC LRRIg1 binding to immobilised human RPTPσ Ig1-FN3, Ig1-3, and Ig1-2. These data demonstrate that the N-terminal three Ig domains of RPTPσ are sufficient for RPTPσ binding to TrkC, although Ig1-2 still retains a reduced affinity interaction. (**b**) SPR analysis of human RPTPσ Ig1-FN3 binding to immobilised mouse TrkC LRRIg2, LRRIg1 and LRR. TrkC Ig1 is necessary for binding of RPTPσ to TrkC. Binding curves (top) and a representative set of sensorgrams (bottom) are illustrated for each interaction. (**c**) Stereo view of Figure 2b (lower panel), illustrating the 1:1 RPTPσ:TrkC interaction mode observed in the chicken RPTPσ Ig1-2:TrkC $LRRIg1_{cryst}$ crystal structure. SigmaA weighted $2F_o$-F_c electron density map (grey) contoured at 1σ and carved at 2 Å around the protein model. (**d**) Mouse TrkC LRRIg1 (red) and human Ig1-FN3 proteins (blue) were incubated alone, or mixed in a 2:1 ratio (purple) prior to SEC-MALS (size-exclusion chromatography-multi-angle light scattering) analysis. Scaled refractive index traces are shown as bold lines and the measured molecular weights as dotted lines. Molecular weight values (with associated uncertainty statistic, provided by the Astra software (Wyatt Technologies)), were measured at the peak of the refractive index traces: mouse TrkC LRRIg1, 45.0 kDa (± 0.2 kDa); human Ig1-FN3, 66.7 kDa (± 0.2 kDa); complex peaks, 97.5 kDa (± 0.2 kDa) and 45.7 kDa (± 0.1 kDa). Theoretical molecular weights: mouse TrkC LRRIg1, 46.3 kDa (assuming 11 N-linked glycans); human RPTPσ Ig1-FN3, 69.4 kDa (assuming 2 N-linked glycans); complex, 115.7 kDa (for a 1:1 stoichiometry).

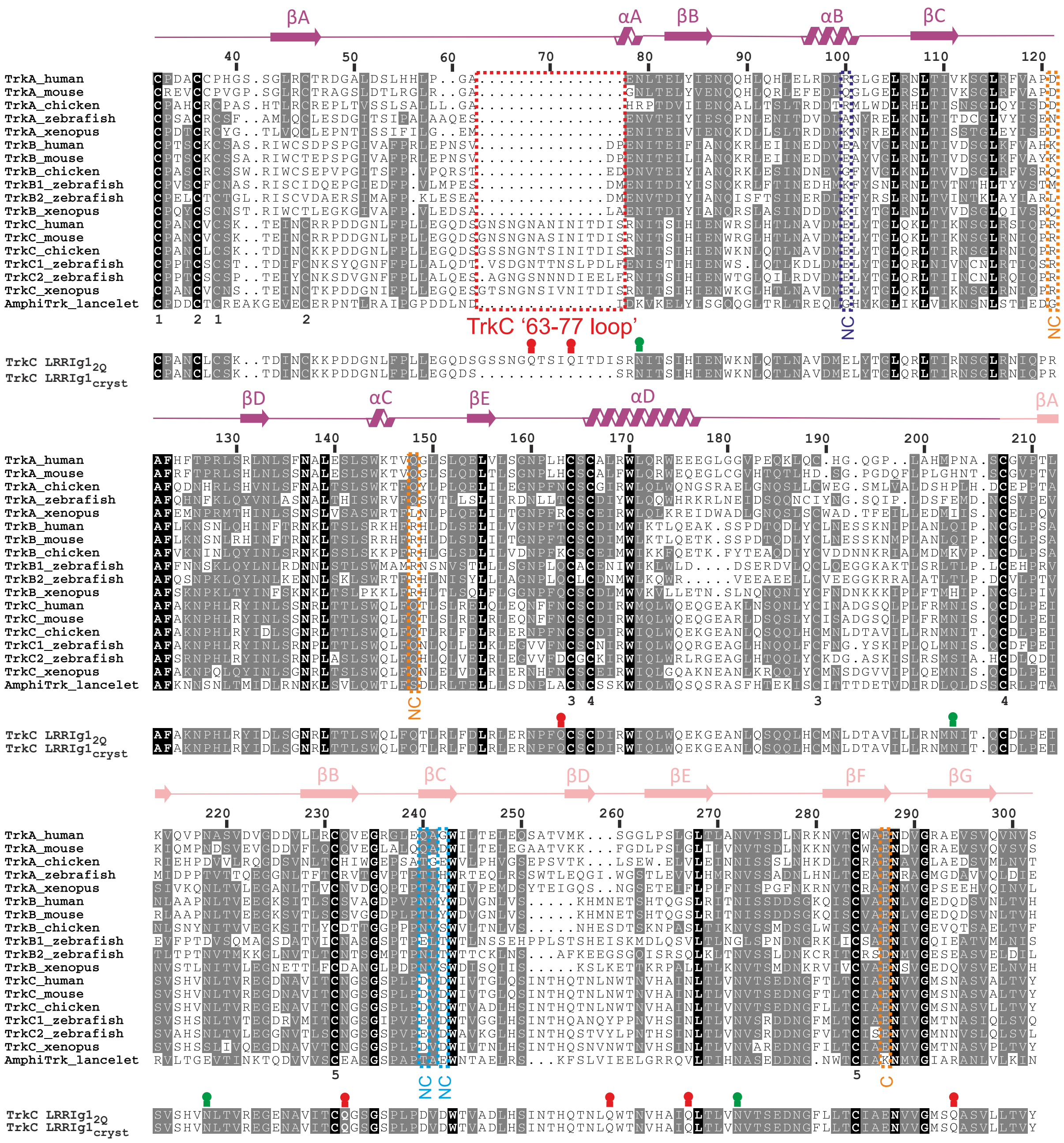

Supplementary Figure 6. Sequence alignment of the N-terminal LRR and Ig1 domains of the Trk family members across species and the TrkC $LRRIg1_{2Q}$ and $LRRIg1_{cryst}$ crystallisation constructs. Amino acid sequences taken from the following sources: TrkA human (NP_001012331.1), mouse (NP_001028296.1), chicken (NP_990709.1), xenopus (XP_002939035.1) and zebrafish (AEX01236.1); TrkB human (AAB33109.1), mouse (NP_001020245.1), chick (NP_990562.1), xenopus (NP_001072653.1) and zebrafish (NP_001184090.1 and XP_003199193.1); TrkC human (AAB33111.1), mouse (NP_032772.3), chicken (NP_990500.1), xenopus (XP_002932277.1) and zebrafish (AEX01237.1 and AEX01238.1); Lancelet AmphiTrk (AAX94284.1). The three mammalian Trks descend from a common pre-vertebrate ancestor AmphiTrk, able to bind all four vertebrate neurotrophin ligands and pre-dating the evolution of complex nervous systems[S3]. Numbers above the sequence alignment correspond to amino acid residue numbers relative to the chicken TrkC sequence (residue 1 is the initial methionine). Secondary structure elements observed for TrkC in the 2.5 Å chicken RPTPσ Ig1-2:TrkC $LRRIg1_{cryst}$ crystal structure, assigned using ksdssp[S1], are shown in magenta (LRR domain) and pink (Ig domain) above the sequence alignment. Numbers below the sequence alignments indicate disulphide bonds. Sticks above the TrkC $LRRIg1_{2Q}$ and TrkC $LRRIg1_{cryst}$ sequences indicate the putative (based on the occurrence of the NXS/T consensus sequence) N-linked glycosylation sites remaining in (green) or removed from (red) these crystallisation constructs. The red dashed box indicates the loop present uniquely in TrkC species, which forms binding site 4 with RPTPσ and is removed from TrkC $LRRIg1_{cryst}$. Residues forming salt bridges with RPTPσ through their side chains at binding sites 1, 2 and 3 are boxed in light blue, orange and dark blue respectively (C and NC denote whether these key residues are conserved or non-conserved between Trk family members). In several TrkB orthologues, N240 (equivalent to D240 in TrkC) harbours a potential glycosylation site which would completely block binding of RPTPσ at binding site 1.

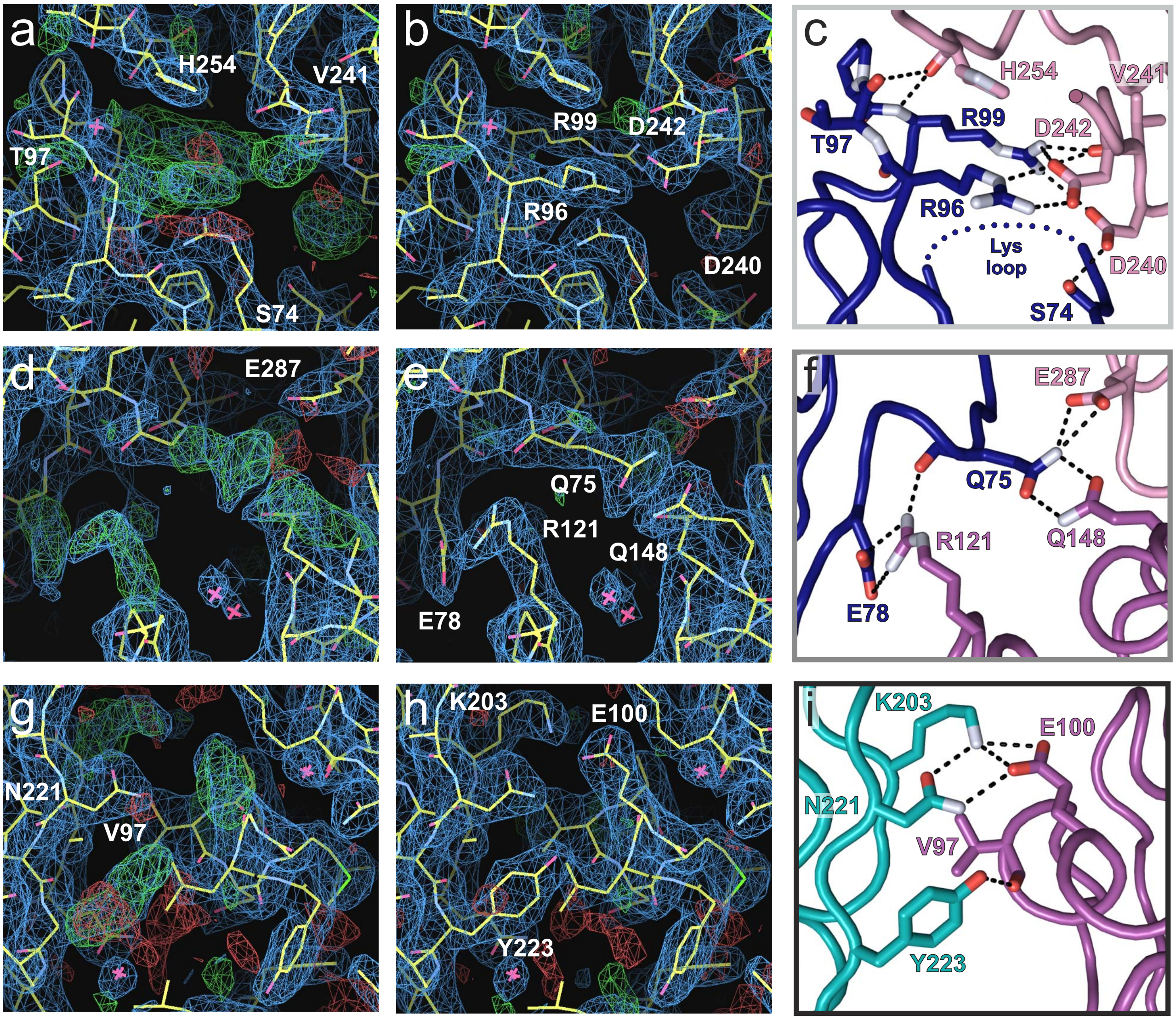

Supplementary Figure 7. RPTPσ:TrkC interaction sites observed in the chicken RPTPσ Ig1-2:TrkC LRRIg1$_{cryst}$ complex crystal structure. (**a-c**) Binding site 1. (a) SigmaA weighted 2F_o-F_c (blue, contoured at 1.5σ) and F_o-F_c (green/red, contoured at +3σ/-3σ) electron density maps following Refmac refinement in the absence of the following sidechains: RPTPσ R96 and R99, TrkC D240 and D242. (b) The same electron density maps and contour levels following refinement in the presence of these sidechains. (c) Final model as in Figure 2c. (**d-f**) Binding site 2. (d) SigmaA weighted 2F_o-F_c (blue, contoured at 1.0σ) and F_o-F_c (green/red, contoured at +3σ/-3σ) electron density maps following Refmac refinement in the absence of the following sidechains: RPTPσ Q75 and E78, TrkC R121 and Q148. (e) The same electron density maps and contour levels following refinement in the presence of these sidechains. (f) Final model as in Figure 2d. (**g-i**) Binding site 3. (g) SigmaA weighted 2F_o-F_c (blue, contoured at 1.0σ) and F_o-F_c (green/red, contoured at +3σ/-3σ) electron density maps following Refmac refinement in the absence of the following sidechains: RPTPσ K203 and Y223, TrkC E100. (h) The same electron density maps and contour levels following refinement in the presence of these sidechains. (i) Final model as in Figure 2e. In panels a,b,d,e,g and h, red crosses represent water molecules. In panels c,f and i, potential electrostatic and hydrogen bonds are indicated with black dashed lines and the disordered RPTPσ Lys-loop by a blue dotted line.

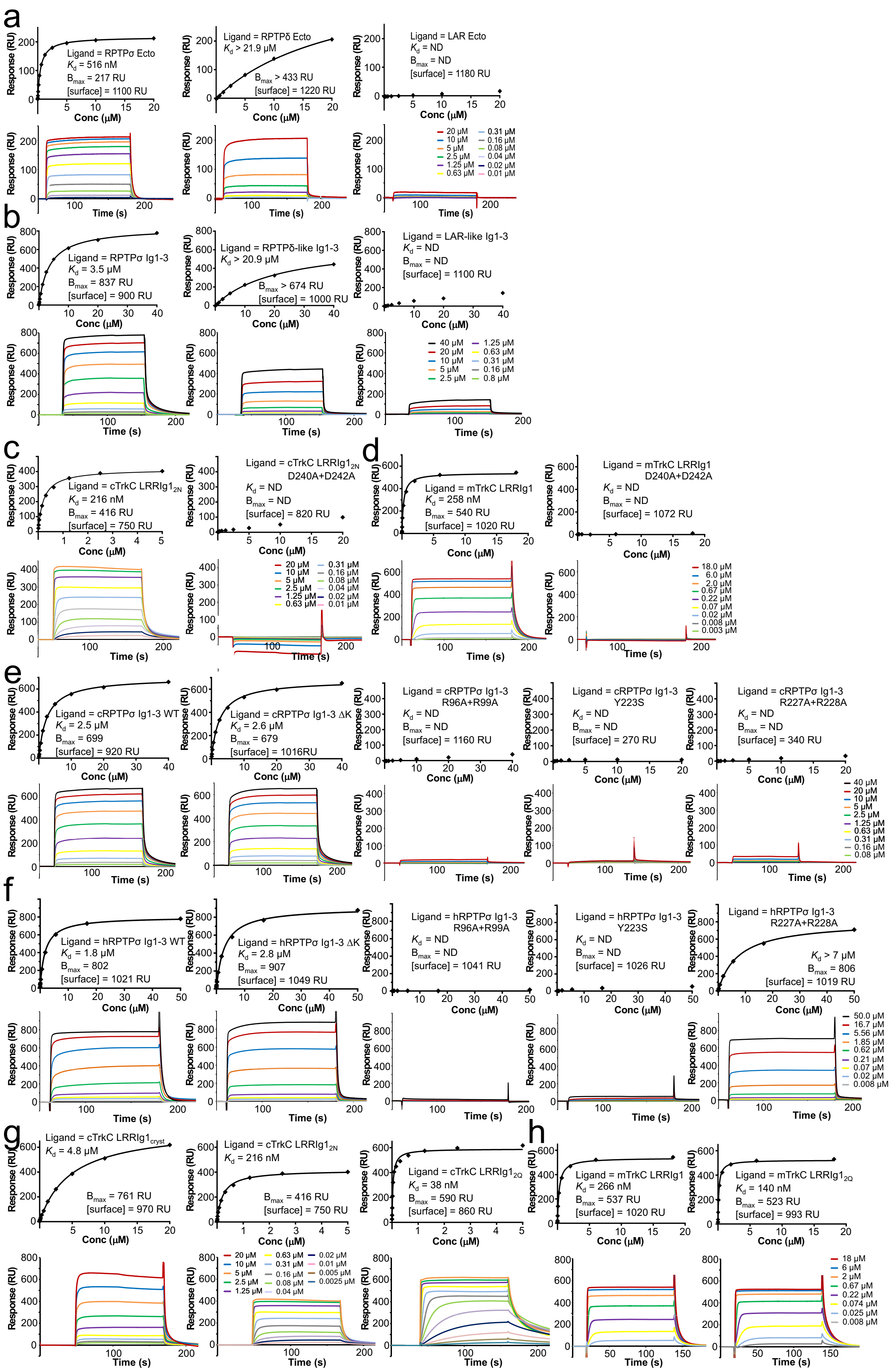
a
Ligand = RPTPσ Ecto
Kd = 516 nM
Bmax = 217 RU
[surface] = 1100 RU
Ligand = RPTPδ Ecto
Kd > 21.9 μM
Bmax > 433 RU
[surface] = 1220 RU
Ligand = LAR Ecto
Kd = ND
Bmax = ND
[surface] = 1180 RU
20 μM
10 μM
5 μM
2.5 μM
1.25 μM
0.63 μM
0.31 μM
0.16 μM
0.08 μM
0.04 μM
0.02 μM
0.01 μM
b
Ligand = RPTPσ Ig1-3
Kd = 3.5 μM
Bmax = 837 RU
[surface] = 900 RU
Ligand = RPTPδ-like Ig1-3
Kd > 20.9 μM
Bmax > 674 RU
[surface] = 1000 RU
Ligand = LAR-like Ig1-3
Kd = ND
Bmax = ND
[surface] = 1100 RU
40 μM
20 μM
10 μM
5 μM
2.5 μM
1.25 μM
0.63 μM
0.31 μM
0.16 μM
0.8 μM
c
Ligand = cTrkC LRRIg1 2N
Kd = 216 nM
Bmax = 416 RU
[surface] = 750 RU
Ligand = cTrkC LRRIg1 2N D240A+D242A
Kd = ND
Bmax = ND
[surface] = 820 RU
d
Ligand = mTrkC LRRIg1
Kd = 258 nM
Bmax = 540 RU
[surface] = 1020 RU
Ligand = mTrkC LRRIg1 D240A+D242A
Kd = ND
Bmax = ND
[surface] = 1072 RU
18.0 μM
6.0 μM
2.0 μM
0.67 μM
0.22 μM
0.07 μM
0.02 μM
0.008 μM
0.003 μM
e
Ligand = cRPTPσ Ig1-3 WT
Kd = 2.5 μM
Bmax = 699
[surface] = 920 RU
Ligand = cRPTPσ Ig1-3 ΔK
Kd = 2.6 μM
Bmax = 679
[surface] = 1016RU
Ligand = cRPTPσ Ig1-3 R96A+R99A
Kd = ND
Bmax = ND
[surface] = 1160 RU
Ligand = cRPTPσ Ig1-3 Y223S
Kd = ND
Bmax = ND
[surface] = 270 RU
Ligand = cRPTPσ Ig1-3 R227A+R228A
Kd = ND
Bmax = ND
[surface] = 340 RU
f
Ligand = hRPTPσ Ig1-3 WT
Kd = 1.8 μM
Bmax = 802
[surface] = 1021 RU
Ligand = hRPTPσ Ig1-3 ΔK
Kd = 2.8 μM
Bmax = 907
[surface] = 1049 RU
Ligand = hRPTPσ Ig1-3 R96A+R99A
Kd = ND
Bmax = ND
[surface] = 1041 RU
Ligand = hRPTPσ Ig1-3 Y223S
Kd = ND
Bmax = ND
[surface] = 1026 RU
Ligand = hRPTPσ Ig1-3 R227A+R228A
Kd > 7 μM
Bmax = 806
[surface] = 1019 RU
50.0 μM
16.7 μM
5.56 μM
1.85 μM
0.62 μM
0.21 μM
0.07 μM
0.02 μM
0.008 μM
g
Ligand = cTrkC LRRIg1 cryst
Kd = 4.8 μM
Bmax = 761 RU
[surface] = 970 RU
Ligand = cTrkC LRRIg1 2N
Kd = 216 nM
Bmax = 416 RU
[surface] = 750 RU
Ligand = cTrkC LRRIg1 2Q
Kd = 38 nM
Bmax = 590 RU
[surface] = 860 RU
h
Ligand = mTrkC LRRIg1
Kd = 266 nM
Bmax = 537 RU
[surface] = 1020 RU
Ligand = mTrkC LRRIg1 2Q
Kd = 140 nM
Bmax = 523 RU
[surface] = 993 RU
Response (RU)
Conc (μM)
Time (s)

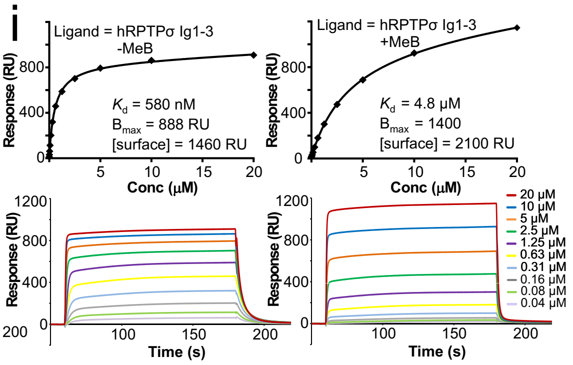

Supplementary Figure 8. Supporting SPR analyses and data:

(**a,b**) Supporting sensograms for Figure 3. (a) Equilibrium SPR experiments testing the binding of the ectodomains of the three human type IIa RPTPs (RPTPσ, RPTPδ and LAR) to immobilised mouse TrkC LRRIg1. Interaction is confirmed to be largely RPTPσ:TrkC specific. (b) SPR equilibrium analyses of chicken TrkC LRRIg1 binding to immobilised chicken RPTPσ Ig1-3, RPTPσ N73S+S74N (RPTPδ-like) Ig1-3 and RPTPσ P97V+T98H (LAR-like) Ig1-3.

(**c-f**) Supporting sensograms for Figure 4. (c) SPR equilibrium analyses of chick RPTPσ Ig1-3 WT binding to immobilised chick TrkC LRRIg1_{2N} (left) and TrkC LRRIg1_{2N} D240A+D242A (right). (d) SPR equilibrium analyses of human RPTPσ Ig1-3 WT binding to immobilised mouse TrkC LRRIg1 WT (left) and D240A+D242A (right). These data illustrate that TrkC binding site 1 is required for the RPTPσ:TrkC interaction. (e) Equilibrium SPR experiments testing the binding of chick TrkC LRRIg1_{2N} to immobilised chick RPTPσ Ig1-3 WT and ΔK, R96A+R99A, Y223S and R227A+R228A mutants. (f) Equilibrium SPR experiments testing the binding of mouse TrkC LRRIg1 WT to immobilised human RPTPσ Ig1-3 WT and ΔK, R96A+R99A, Y223S and R227A+R228A mutants. These data demonstrate that RPTPσ binding sites 1,3 and 4 are all required for the RPTPσ:TrkC interaction.

(**g,h**) Supporting sensograms for Figure 5. (g) Equilibrium SPR experiments testing the binding of chicken RPTPσ Ig1-3 to immobilised chicken TrkC LRRIg1 excluding residues 63-77 (chick TrkC LRRIg1_{cryst}), with these residues reinserted (chick TrkC LRRIg1_{2N}) or a N68Q+N72Q variant, designed to lack two putative N-glycans (chick TrkC LRRIg1_{2Q}). All three TrkC constructs have N163Q, N232Q, N259Q, N267Q and N294Q mutations to remove N-linked glycosylation sites which lie remotely from the RPTPσ:TrkC binding interface. Removal of residues 63-77 from TrkC is seen to decrease the binding affinity for RPTPσ by 20-fold (K_d 216 nM vs 4.8 μM), supporting the involvement of this loop region in TrkC:RPTPσ binding site 4 as suggested by the crystal structure. (h) Equilibrium SPR experiments testing the binding of human RPTPσ Ig1-3 to immobilised mouse TrkC LRRIg1 (WT) and mouse TrkC LRRIg1_{2Q}. A comparable binding constant is observed between chick RPTPσ:chick TrkC and human RPTPσ:mouse TrkC (K_d 216 nM vs 266 nM) when the loop is present, and removal of N-linked glycans increases the strength of interaction in both cases, though less so for the mammalian interaction pair (new K_d 38 nM vs 140 nM).

(**i**) Influence of RPTPσ mini-splice exon meB upon interaction with TrkC. SPR equilibrium analyses of mouse TrkC LRRIg1 binding to immobilised human RPTPσ Ig1-3 with (left) and without (right) insertion of meB, four amino acid residues ELRE between R225 and V226. This data demonstrates that addition of this exon into the RPTPσ Ig2-3 linker present at binding site 4, partially reduces the affinity of RPTPσ:TrkC binding.

Binding curves (top) and a representative set of sensograms (bottom) are illustrated for each interaction. The legends provided apply to all sets of sensograms within a given panel. As in all text, RPTPσ residue numbering is relative to the chicken protein, mouse RPTPσ would be +1.

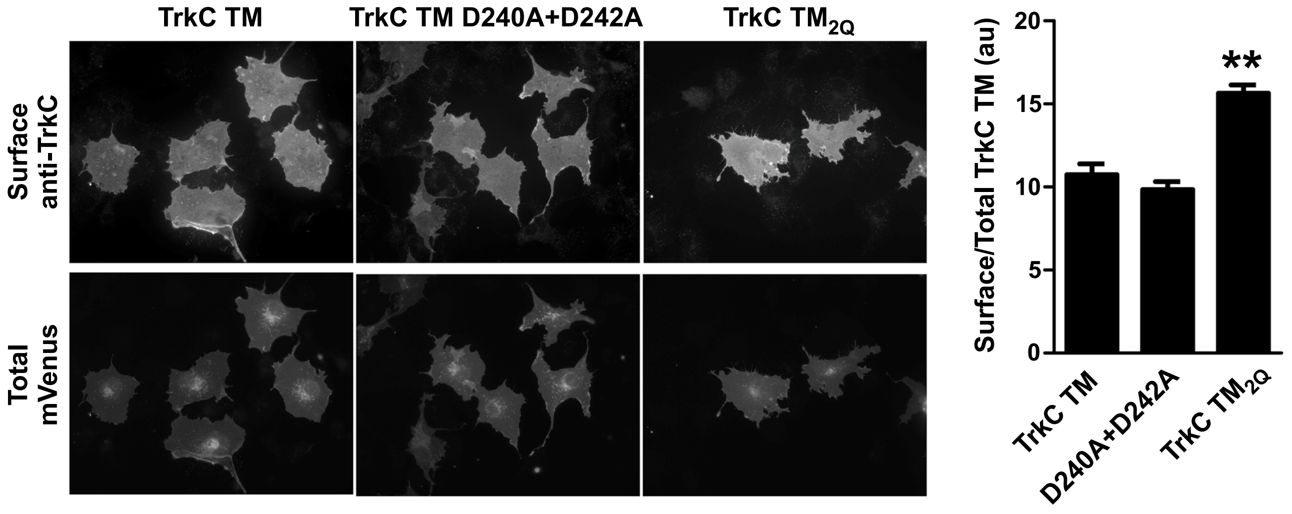

Supplementary Figure 9. Relative cell surface expression levels of TrkC TM constructs. COS-7 cells were transfected with mVenus-tagged TrkC TM (WT), TrkC TM D240A+D242A and TrkC TM_{2Q}. Surface expression was visualized by immunofluorescence with anti-TrkC antibody without membrane permeabilization (top row). Total expression was indicated by the expression of mVenus (bottom row). Surface trafficking was enhanced for the 63-77 loop mutant and unchanged for the D240A+D242A mutant as compared with wild-type TrkC TM. ANOVA p <0.0001, **p<0.001 compared with TrkC TM by posthoc Bonferroni's multiple comparison test, n = 15-21 cells.

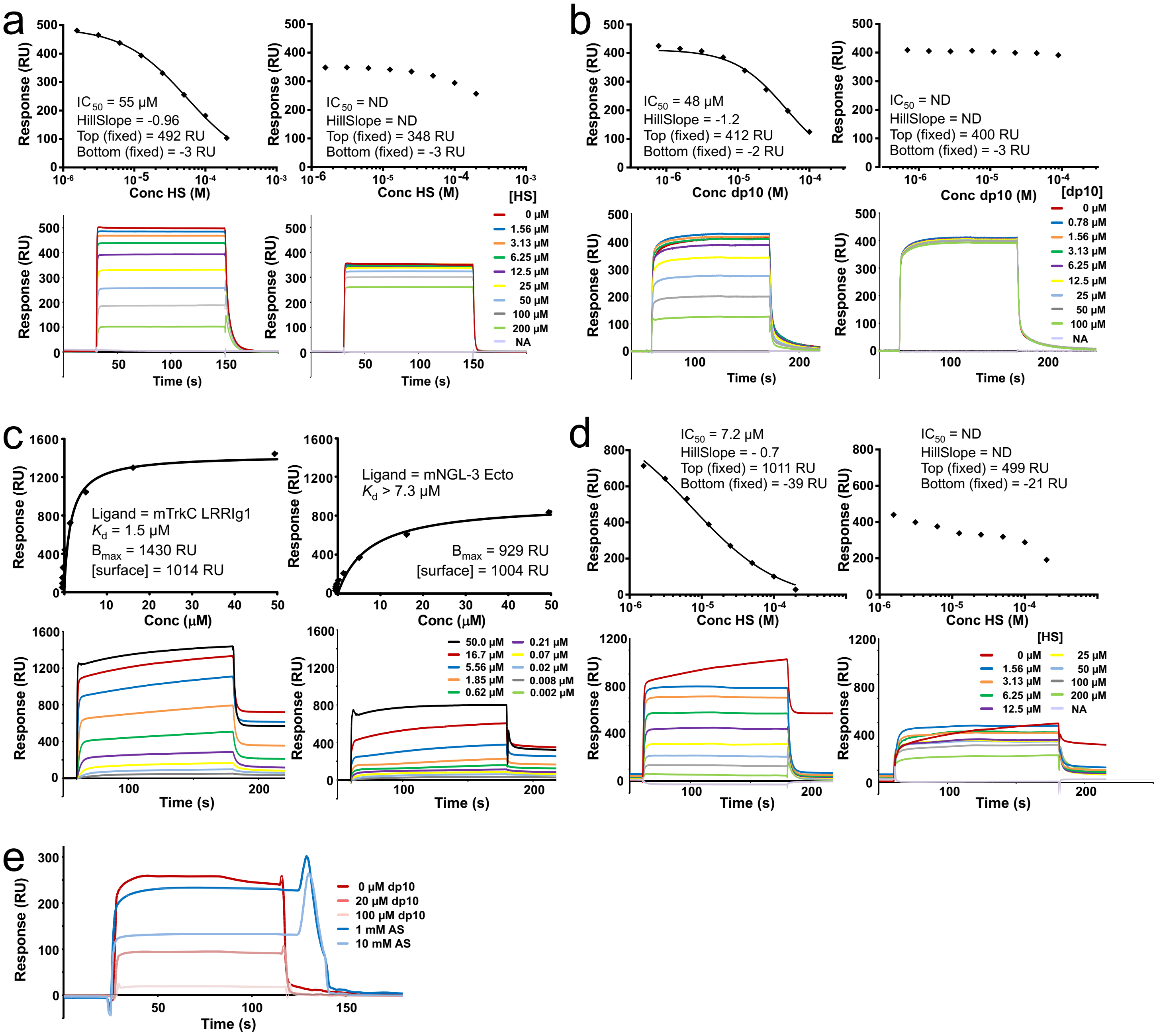

Supplementary Figure 10. Specificity of GAG-mediated inhibition of RPTPσ-TrkC binding. (**a**) Equilibrium SPR experiments testing the binding of 10 μM human RPTPσ Ig1-3 (left) and 10 μM human RPTPσ Ig1-3 ΔK (right) to immobilised mouse TrkC-LRRIg1 in the presence of increasing concentrations of heparan sulfate (HS). (**b**) Binding of 5 μM chick TrkC LRRIg1$_{2N}$ to immobilised chick RPTPσ Ig1-3 WT (left) and ΔK (right) in the presence of increasing concentrations of heparin-dp10. In contrast to wildtype RPTPσ, the ΔK mutant is insensitive to dp10 addition, and retains full binding to TrkC. (**c**) Binding of human RPTPσ sEcto to immobilised mouse TrkC LRRIg1 or mouse NGL-3 Ecto. Binding curves (top) and a representative set of sensorgrams (bottom) are illustrated for each interaction. (**d**) Binding of 10μM human RPTPσ sEcto to immobilised mouse TrkC LRRIg1 or mouse NGL-3 Ecto in the presence of increasing concentrations of HS. (a, b and d) non-linear regression dose-response curves were fitted to the data (allowing a variable Hill Slope, but fixing top and bottom values based on experimental controls measured in the absence of HS/dp10 or in the absence of both analyte (NA) and HS/dp10 (0 μM) respectively). In contrast to mouse TrkC, the interaction of mouse NGL-3 with RPTPσ appears relatively insensitive to HS addition. (**e**) High concentrations of a charged salt, ammonium sulphate (up to 10 mM) were unable to inhibit chick RPTPσ:TrkC binding to a comparable or greater extent than 500-fold less concentrated dp10 (20 μM).

Supplementary Table 1 Summary of all equilibrium SPR experiments

**RPTPσ-TrkC binding experiments**			
Protein construct 1 (immobilised ligand)	Protein constuct 2 (analyte)	Equilibrium binding constant K_d (μM)	Related Figure
**RPTP**	**TrkC**		
Human RPTPσ Ig1-FN3	Mouse LRRIg1	0.468	Supp. Fig. 5a
Human RPTPσ Ig1-3	Mouse LRRIg1	0.551	
Human RPTPσ Ig1-2	Mouse LRRIg1	2.4	
Human RPTPσ Ecto	Mouse LRRIg1	0.516	Fig. 3b Supp. Fig. 8a
Human RPTPδ Ecto	Mouse LRRIg1	>21.9	
Human LAR Ecto	Mouse LRRIg1	ND	
Chicken RPTPσ Ig1-3	Chicken LRRIg1	3.5	Fig. 3c Supp. Fig. 8b
Chicken RPTPδ-like Ig1-3	Chicken LRRIg1	>20.9	
Chicken LAR-like Ig1-3	Chicken LRRIg1	ND	
Chicken RPTPσ Ig1-3 WT	Chicken LRRIg1_{2N}	2.5	Fig. 4c Supp. Fig. 8e,f
Chicken RPTPσ Ig1-3 ΔK	Chicken LRRIg1_{2N}	2.6	
Chicken RPTPσ Ig1-3 R96A+R99A	Chicken LRRIg1_{2N}	ND	
Chicken RPTPσ Ig1-3 Y223S	Chicken LRRIg1_{2N}	ND	
Chicken RPTPσ Ig1-3 R227A+R228A	Chicken LRRIg1_{2N}	ND	
Human RPTPσ Ig1-3 WT	Mouse LRRIg1	1.8	
Human RPTPσ Ig1-3 ΔK	Mouse LRRIg1	2.8	
Human RPTPσ Ig1-3 R96A+R99A	Mouse LRRIg1	ND	
Human RPTPσ Ig1-3 Y223S	Mouse LRRIg1	ND	
Human RPTPσ Ig1-3 R227A+R228A	Mouse LRRIg1	>7	
Human RPTPσ Ig1-3 –MeB	Mouse LRRIg1	0.58	Supp. Fig. 8i
Human RPTPσ Ig1-3 +MeB	Mouse LRRIg1	4.8	
**TrkC/NGL-3**	**RPTP**		
Mouse TrkC LRRIg2	Human RPTPσ Ig1-FN3	0.202	Supp. Fig. 5b
Mouse TrkC LRRIg1	Human RPTPσ Ig1-FN3	0.278	
Mouse TrkC LRR	Human RPTPσ Ig1-FN3	ND	
Chicken TrkC LRRIg1_{2N}	Chicken RPTPσ Ig1-3	0.216	Fig. 4b Supp. Fig. 8c,d
Chicken TrkC LRRIg1_{2N} D240A+D242A	Chicken RPTPσ Ig1-3	ND	
Mouse TrkC LRRIg1	Mouse RPTPσ Ig1-3	0.258	
Mouse TrkC LRRIg1 D240A+D242A	Mouse RPTPσ Ig1-3	ND	
Chicken TrkC LRRIg1_{cryst}	Chicken RPTPσ Ig1-3	4.8	Fig. 5a Supp. Fig. 8g,h
Chicken TrkC LRRIg1_{2N}	Chicken RPTPσ Ig1-3	0.216	
Chicken TrkC LRRIg1_{2Q}	Chicken RPTPσ Ig1-3	0.038	
Mouse TrkC LRRIg1	Mouse RPTPσ Ig1-3	0.266	
Mouse TrkC LRRIg1_{2Q}	Mouse RPTPσ Ig1-3	0.140	
Mouse TrkC LRRIg1	Human RPTPσ sEcto	1.5	Supp. Fig. 10c
Mouse NGL-3 Ecto	Human RPTPσ sEcto	>7.3	

**GAG competition experiments**			
Protein construct 1 (immobilised ligand)	Protein constuct 2 + GAG (analyte)	IC_{50} for GAG inhibition (μM)	Related Figure
Mouse TrkC LRRIg1	Human RPTPσ Ig1-3 +HS	55	Fig. 6a Supp. Fig. 10a,b
Mouse TrkC LRRIg1	Human RPTPσ Ig1-3 ΔK + HS	ND	
Chicken RPTPσ Ig1-3	Chicken TrkC LRRIg1_{2N} + dp10	48	
Chicken RPTPσ Ig1-3 ΔK	Chicken TrkC LRRIg1_{2N} + dp10	ND	
Mouse TrkC LRRIg1	Human RPTPσ sEcto + HS	7.2	Supp. Fig. 10d
Mouse NGL3 Ecto	Human RPTPσ sEcto + HS	ND	

Additional references for supplementary figures

S1 Kabsch, W. & Sander, C. Dictionary of protein secondary structure: Pattern recognition of hydrogen-bonded and geometrical features. *Biopolymers* **22**, 2577-2637, (1983).

S2 Luçic´, V., Yang, T., Schweikert, G., Förster, F. & Baumeister, W. Morphological Characterization of Molecular Complexes Present in the Synaptic Cleft. *Structure* **13**, 423-434, (2005).

S3 Benito-Gutiérrez, È., Nake, C., Llovera, M., Comella, J. X. & Garcia-Fernàndez, J. The single AmphiTrk receptor highlights increased complexity of neurotrophin signalling in vertebrates and suggests an early role in developing sensory neuroepidermal cells. *Development* **132**, 2191-2202, (2005).
